# Major Contribution of Flowering Time and Vegetative Growth to Plant Production in Common Bean As Deduced from a Comparative Genetic Mapping

**DOI:** 10.3389/fpls.2016.01940

**Published:** 2016-12-26

**Authors:** Ana M. González, Fernando J. Yuste-Lisbona, Soledad Saburido, Sandra Bretones, Antonio M. De Ron, Rafael Lozano, Marta Santalla

**Affiliations:** ^1^Grupo de Biología de Agrosistemas, Misión Biológica de Galicia-Consejo Superior de Investigaciones CientificasPontevedra, Spain; ^2^Departamento de Biología y Geología (Genética), Centro de Investigación en Biotecnología Agroalimentaria, Universidad de AlmeríaAlmería, Spain; ^3^Somos Semilla, Seed LibraryGuanajuato, Mexico

**Keywords:** *Phaseolus vulgaris* L., flowering time, vegetative growth, rate of plant production, quantitative trait locus

## Abstract

Determinacy growth habit and accelerated flowering traits were selected during or after domestication in common bean. Both processes affect several presumed adaptive traits such as the rate of plant production. There is a close association between flowering initiation and vegetative growth; however, interactions among these two crucial developmental processes and their genetic bases remain unexplored. In this study, with the aim to establish the genetic relationships between these complex processes, a multi-environment quantitative trait locus (QTL) mapping approach was performed in two recombinant inbred line populations derived from inter-gene pool crosses between determinate and indeterminate genotypes. Additive and epistatic QTLs were found to regulate flowering time, vegetative growth, and rate of plant production. Moreover, the pleiotropic patterns of the identified QTLs evidenced that regions controlling time to flowering traits, directly or indirectly, are also involved in the regulation of plant production traits. Further QTL analysis highlighted one QTL, on the lower arm of the linkage group Pv01, harboring the *Phvul.001G189200* gene, homologous to the *Arabidopsis thaliana TERMINAL FLOWER1* (*TFL1*) gene, which explained up to 32% of phenotypic variation for time to flowering, 66% for vegetative growth, and 19% for rate of plant production. This finding was consistent with previous results, which have also suggested *Phvul.001G189200 (PvTFL1y*) as a candidate gene for determinacy locus. The information here reported can also be applied in breeding programs seeking to optimize key agronomic traits, such as time to flowering, plant height and an improved reproductive biomass, pods, and seed size, as well as yield.

## Introduction

Common bean (*Phaseolus vulgaris* L.) is the most important food legume for direct human consumption. It is considered to be a rich source of proteins, micronutrients, and calories for human daily needs (Broughton et al., 2003). It is grown over a wide range of latitudes; however, there is an adaptation of each cultivar to a relatively narrow range of latitudes. Wild bean forms present indeterminate growth habits and require day-lengths of <12 h to initiate flowering. Together, earlier flowering and determinate growth habit genotypes under day-lengths longer than 12 h allowed for the adaptation to higher latitudes (Gepts and Debouck, 1991). Domesticated and wild common bean display notable differences in growth habit types. Thus, there is a considerable variability in domesticated cultivars, which show pronounced differences in growth form, i.e., determinate vs. indeterminate, and growth habit, i.e., I, II, III, and IV (Debouck and Hidalgo, 1986; Debouck, 1991). Determinate common bean cultivars generally flower and mature early, and the transition of the terminal shoot meristem from vegetative to reproductive state results in a terminal inflorescence in the axil of the older leaf primordia. By contrast, in indeterminate cultivars, the terminal shoot meristem continuously produces modular units until senescence, each one consisting of a leaf and an inflorescence. Thus, the plant will have a terminal shoot meristem that remains in a vegetative state throughout the production of vegetative and reproductive structures (Ojehomon and Morgan, 1969; Tanaka and Fujita, 1979). It has been documented that stem termination has great effects on plant height, flowering and maturity period, amount of branching, length of internodes on the main stem, and node production, which conditions how many flowers and leaves, and therefore pods and seeds, are produced. Thus, understanding the genetic control of vegetative growth and flowering time in common bean will enable genetic manipulation of major components of yield.

Previous studies demonstrated that the *FIN* locus mainly regulates stem growth habit in common bean and that the indeterminate growth habit is dominant over the determinate one. This gene was mapped on the Linkage Group (LG) Pv01 (Norton, 1915; Koinange et al., 1996). Despite the fact that *FIN* is a monogenic locus, it is possible to find a wide range of stem termination types among common bean cultivars, which may be regulated by a second unnamed locus mapped on Pv07 (Kolkman and Kelly, 2003). In addition, control of twining has been attributed to the *TOR* gene, distinct from the *FIN* locus (Norton, 1915) although either *FIN* has a pleiotropic effect on twining or that *TOR* is tightly linked to *FIN*. Additionally, other loci have been reported to control flowering time and other flowering-related traits in common bean (Norton, 1915; Wallace et al., 1993; Jung et al., 1996; Bassett, 1997; McClean et al., 2002; Tar'an et al., 2002; Kolkman and Kelly, 2003; Blair et al., 2006; Checa and Blair, 2008; Chavarro and Blair, 2010; Pérez-Vega et al., 2010). The majority of these loci have been detected as Quantitative Trait Loci (QTL), although they have been treated as Mendelian factors in some studies. Probably, different works have detected the same loci; however, the lack of common markers among different mapping studies makes difficult to determine whether or not they are the same locus.

During the past two decades, the model species *Arabidopsis thaliana* has been mainly used to study the process of phase transition from vegetative to reproductive growth at developmental, environmental, genetic, and molecular levels (Weigel, 1995; Yanofsky, 1995; Bradley et al., 1997; Ma, 1998; Pidkowich et al., 1999). In this species, floral meristem identity is determined by two different pathways. The heterodimer FLOWERING LOCUS T (FT)/FLOWERING LOCUS D (FD) is proposed to promote flowering initiation through activating the *APETALA1* (*AP1*) expression (Abe et al., 2005; Wigge et al., 2005). Furthermore, another key regulator of flowering initiation is *TERMINAL FLOWER1* (*TFL1*), a floral repressor and a regulator of inflorescence meristem development, which acts by repressing the expression of *AP1* and *LEAFY* (*LFY*) floral identity genes (Bradley et al., 1997; Ohshima et al., 1997; Nilsson et al., 1998; Boss et al., 2004). FT and TFL1 have closely related sequences, although key amino acids have been found, which have been proposed as responsible for making that these two proteins perform opposite functions. Within the legumes, the value of a comparative approach to candidate gene identification has led to the characterization of the molecular identity of *TFL1* co-orthologs such as *FIN* in common bean (designated as *PvTFL1y*; Repinski et al., 2012); *Dt1* in soybean (*GmTFL1*; Tian et al., 2010); and *DETERMINATE* (*DET*; *PsTFL1a*) and *LATE FLOWERING* (*LF*; *PsTFL1c*) in pea (Foucher et al., 2003; Weller and Ortega, 2015). Recent findings have clearly shown that in several legume species, determinate inflorescence architecture is conferred by mutation of specific *TFL1* genes (Benlloch et al., 2015). The determinate growth habit caused by mutations within specific *TFL1*-homologs in other grain legumes indicates that the determinate function is conserved in these species (Kong et al., 2010; Tian et al., 2010; Kwak et al., 2012; Repinski et al., 2012; Dhanasekar and Reddy, 2014).

In this study, the aim was to identify the genetic determinants of vegetative growth and its relationship to days to flowering and fruit maturation in two Recombinant Inbred Line (RIL) populations by using a mixed-model based composite mapping method for QTL detection. Both populations were derived from inter-gene (Andean × Mesoamerican) pool crosses between determinate and indeterminate genotypes, and shared an Andean determinate common parent, which allowed for the comparison of the results in reference to a tester line. Parents of each RIL population also differ in rate of plant production (leaf, flower and fruit size, and yield components), allowing for the dissection of the genetic architecture of these traits, as well as the study of the relationship among vegetative growth and these traits. Comparative QTL mapping indicated that, in both RIL mapping populations, the genomic region where the *FIN* locus is located not only is involved in the regulation of vegetative growth and rate of plant production but also affect flowering time, suggesting a pattern of pleiotropic effects accounting for the genetic bases of these traits.

## Materials and methods

### Plant material and trials

**Figure 1:**
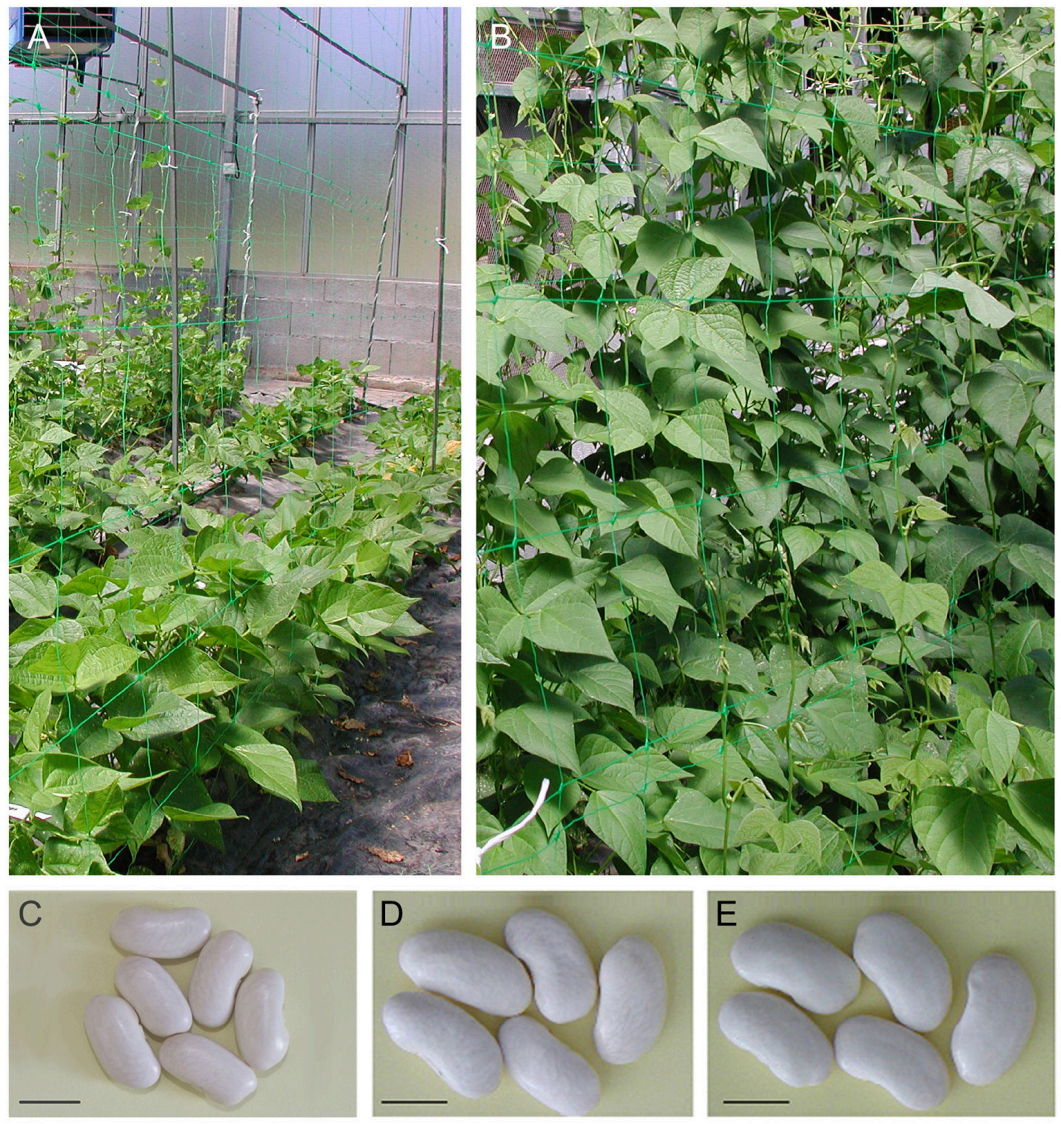
**Determinate type I and indeterminate type IV growth habits, and dry seeds of the parental genotypes Beluga (A,C)**, PHA0399 **(B,D)**, and PHA0419 **(B,E)**. Scale bar 1 cm.

Two F_2:8_ RIL populations derived by single-seed descent from an F_2_ population from crosses between the Mesoamerican (M) and Andean (A) gene pools were used. The MA population was generated from the cross between lines PHA0419 (Mesaomerican) and Beluga (Andean), whereas the AM population was obtained from the Beluga and PHA0399 (Mesoamerican) cross. Beluga is a large white kidney seed which seed weight averages 60 g 100 seeds^−1^ (range: 52–66 g 100 seeds^−1^), 55 cm in length of main stem with a type I determinate growth habit, and blooms 30 days after planting (DAP). Beluga is resistant to bean common mosaic virus (BCMV), as it bears the autosomal dominant hypersensitive *I* gene, and possesses the Andean *Co-1* gene for resistance to races 65 and 73 of anthracnose (Kelly et al., 1999). Both PHA0419 and PHA0399 are great northern beans which averages 86 g 100 seeds^−1^ (range: 76–103 g 100 seeds^−1^), and shared a type IV climbing indeterminate growth habit (averages 278 cm of length of main stem) and late flowering (46 DAP as average). PHA-0419 possesses the Mesoamerican *Co-4*^2^, *Co-6*, and *Co-10* genes that condition resistance to races 23, 39, 102, 448, and 1545 of anthracnose, while PHA-0399 carries *Co-4*^2^, *Co-4*^3^, and *Co-6* genes that condition resistance to races 23, 55, 102, and 1545 of anthracnose (Santalla et al., 2004; Figure 1). RILs and parents were evaluated in open field (F) and greenhouse (G) environments in Pontevedra (Spain, 42°24′ N, 8°38′ W, 40 masl). Briefly, the RILs populations and the parents were planted between April and May in 2008 and 2009 under field conditions: F108 planted on 2nd April 2008 (93 Julian days and day-length ~12.4 h) and F109 planted on 8th May 2009 (128 Julian days and day-length ~11.1 h). RIL and parental lines were also planted between February and March in 2009 and 2008 under greenhouse conditions: G108 planted on 25th March 2008 (85 Julian days and day-length ~12.2 h) and G109 planted on 9th February 2009 (40 Julian days and day-length ~10.1 h). Field and greenhouse experiments were designed according to a complete randomized block design with two replications. Each genotype was planted in a single row plot, 0.80 m apart and 3.0 m long with a total of 15 plants per row; the density was 30,000 plants/ha. Crop management was in accordance with common bean local practices.

### Field evaluation and data collection

Data were collected for time to flowering and fruit maturity evaluated as days to flowering, days to immature pod harvest, and days to physiological pod maturity (Supplementary Table 1). The days to flowering (FT) trait was scored as the number of days from planting date to the opening of the first flower. The days to young-green pod (PGT) trait was recorded when a plant had 50% of the immature pods. The days to physiological pod maturity (PST) trait was scored as the number of days from planting date to the appearance of a mature dry pod on a primary branch.

Measurements were collected for growth ability reordered as length of main stem, number of primary stem branches, and internode length, in five random plants from each RIL. Length of main stem (LMS) was measured in cm from the base of the plant to the uppermost leaflet on the longest branch. The number of primary stem branches (NPB) was recorded as the number of the stems winding around the support strings at the midpoint of the plant length. Internode length (LI) was recorded at the fifth internode on the main stem in cm.

To quantify crop growth and productivity, flower, pod and seed size, number of seeds per pod, number of pods per plant, and seed yield were determined in ten random flowers and fruits on a plot basis. Bracteole length (BL) measured in mm along the midrib of the lamina. Bracteole width (BWI) recorded in mm between the widest lobes of the lamina perpendicular to the lamina mid-rib. Maximum leaflet width (LWI) was measured in cm at the largest point perpendicular to the midrib. Leaflet length (LL) was recorded in mm from the lamina tip to the point of petiole intersection along the midrib. Pod length (PL) was recorded in mm as the exterior distance from the peduncle connection point to the apex excluding the beak. Pod width (PWI) determined in mm as the distance at right angles to the sutures at the level of the second seed from the apex. Pod thickness (PT) was recorded in mm as the distance between sides at the level of the second and third seed from the apex. Seed width (SWI) was the longest distance across the hilum, in mm. Seed thickness (ST) was measured in mm from hilum to opposite side. Seed length (SL) was measured in mm parallel to the hilum. Seed weight (SW) was determined on 100 dry seeds per plot. The number of seeds per pod (NSP), number of pods per plant (NPP), and seed yield (SY) were recorded and expressed in kilograms per hectare at moisture content of 140 g kg^−1^.

### Experimental design and statistical data analysis

Comprehensive statistical analysis (mean value, standard error, and range of variation) and normality test (Kolmogorov-Smirnov test) were carried out for each quantitative trait and environment. LMS, NPB, LI, BL, BWI, SW, NSP, NPP, and SY traits failed to meet normality assumptions and the Box-Cox transformation was used to improve normality, while LOG transformation was used for LL and LWI traits. Significant variation in the expression of traits through the environment conditions was analyzed using PROC MIXED (SAS Institute Inc. 9.04, Cary, NC, USA). The estimates of variance components were obtained by the REML method with Proc MIXED in SAS9.04 and used to calculate the broad-sense heritability on a progeny-mean basis (*h*^2^ = $\sigma_{\lambda }^{2}$/[($\sigma_{t}^{2}$/*e*) + $\sigma_{\lambda }^{2}+$ ($\sigma_{e}^{2}$/*re*)] where: $\sigma_{\lambda }^{2}=$ genetic variance of the trait; $\sigma_{t}^{2}=$ variance due to environmental factors; $\sigma_{e}^{2}$ = error variance; *r* = number of replications and *e* = number of environments). In order to increase the precision of the entry mean basis heritability estimate, it was used the harmonic means for the number of replications and environments, where each experimental line was tested (Holland et al., 2003). Approximate standard errors of heritability estimates were obtained by the delta method (Holland, 2006). Phenotypic Pearson correlation coefficients for the different traits were calculated using PROC CORR through the environment conditions in SAS9.04.

### DNA isolation and marker analysis

Young leaves from individual plants of both RIL mapping populations (60 and 179 lines for AM and MA, respectively) were collected for genomic DNA isolation as described by Chen and Ronald (1999). Total DNA was stored in sterile water and examined by electrophoresis in 1% agarose gels in 1X SB buffer (10 mM sodium boric acid). The concentration and quality of extracted DNA was determined by reading at 230, 260, and 280 nm using Nanodrop Thermo ScientificTM (NanoDrop 2000). DNA was diluted in sterile water to get a working dilution of 5–10 ng/μL, which was used for PCR analysis.

A set of 634 markers [Simple Sequence Repeats (SSR) and single nucleotide polymorphisms (SNP)] were tested for polymorphisms in the parental genotypes. SSR markers were named according to the respective authors [IAC: Benchimol et al., 2007; Cardoso et al., 2008; Campos et al., 2011; BM, GATS: Gaitán-Solís et al., 2002; Blair et al., 2009a; BMb: Córdoba et al., 2010; BMc: Blair et al., 2009b, 2011; BMd: Blair et al., 2003; PV, Pvtttc001: Yu et al., 2000; PvBR: Buso et al., 2006; Grisi et al., 2007; PVEST, X04001: García et al., 2011; PvM: Hanai et al., 2007]. SSR markers were evaluated either by gel electrophoresis or capillary electrophoresis in an ABI PRISM® 3130 XL Genetic Analyzer (Applied Biosystems, USA). SNP markers were analyzed by High Resolution Melting Technology (HRM) using a LightScanner instrument (Idaho Technology), according to the protocols described by Montgomery et al. (2007). These markers were designated as Leg- (Hougaard et al., 2008) and SNP- (McConnell et al., 2010), respectively.

### Linkage map and QTL analysis

JoinMap 4.0 software (van Ooijen, 2006) was used to construct the genetic linkage maps for both MA and AM mapping populations. A minimum logarithm of odds ratio (LOD) of 6.0 was considered to establish significant linkage. Locus order within the LOD grouping was generated for each LG using the regression mapping algorithm with the following JoinMap parameters: Rec = 0.3, LOD = 2.0, and Jump = 5. The Kosambi map function (Kosambi, 1944) was used to calculate the genetic distance between markers. LGs were designated according to Pedrosa-Harand et al. (2008). JoinMap 4.0 (van Ooijen, 2006) was also used to generate pairwise recombination frequencies and LOD scores for the selected sets of representative loci for each LG, which were then combined into a single group node in the navigation tree. The regression mapping algorithm was used and the LG lengths for the consensus map of all the representative markers were calculated.

The physical position of genetic markers was obtained by sequence similarity analysis using BLASTN (Altschul et al., 1997) against the common bean genome (Phytozome v.11: Pv1.0; Schmutz et al., 2014) in the Phytozome database (http://www.phytozome.net/). The correlations between physical distance and genetic map in each LG were calculated by Spearman's rank correlation coefficients.

QTLNetwork 2.0 software (Yang et al., 2008) was used for multi-environment QTL analyses. In order to identify putative single-locus QTLs and their environment interactions (QTLs × Environment, QE), a mixed-model based composite interval mapping method (MCIM) was carried out for one-dimensional genome scan. In addition, with the aim to detect epistatic QTLs (E-QTL) and their environment interaction effects (E-QTLs × Environment, E-QE), a two-dimensional genome scan was performed. A QTL was declared significant when the *F-value* was higher than the *F-value* threshold determined by a 1000 permutation test at 95% confidence level. Markov Chain Monte Carlo method was used to estimate the effects of QTLs and environment interactions (Wang et al., 1994). Both testing and filtration window sizes were set at 10 cM, with a walk speed of 1 cM. Candidate interval selection, putative QTL detection, and QTL effect was estimated with an experimental-wise significance level of 0.05. MapChart 2.2 software (Voorrips, 2002) was used to draw the genetic map and the detected QTLs. QTL regions were positioned onto the consensus map. QTL designations were made using abbreviations for the quantitative trait, and followed by LG number at which the QTL was mapped.

## Results

### Vegetative growth and time to flowering variation

Genetic variation for vegetative growth as length of main stem and rate of plant production has been studied, together with its relationship to days to flowering and maturity in both inter-gene pool RIL populations. The populations were grown in different years and locations (field vs. greenhouse). Both populations segregated for different levels of growth ability: indeterminate vs. determinate growth habits. Classification of the 60 and 179 lines of AM and MA RIL populations for growth habit identified 37 and 63 lines as homozygous determinate type I, and 23 and 116 lines as homozygous indeterminate type IV, respectively. The observed growth habit segregation fitted to a 1:1 ratio (χ^2^ = 3.27, *P* ≤ 0.05) for the AM population, indicating that a single gene determined the trait. However, growth habit distribution appeared distorted in the MA population (χ^2^ = 15.69, *P* ≥ 0.05). On the basis of segregation analysis results, the gene for growth habit (*FIN*) was mapped along with the molecular markers. Supplementary Figure 1 shows phenotypic distribution of the RIL populations based on line means. The large range of variation and transgressive segregations observed for most traits in both RIL populations suggested a complex control of these traits, with positive alleles shared between the two parents of the RIL populations. Transgressive segregation in both directions was observed for days to flowering and maturity in both populations. For LMS, bimodality was observed in the AM population, with a clear separation of phenotypic classes that would indicate monogenic inheritance, although this separation was not evident in the MA distribution. Lines shorter or taller than the height of the parents were found for LMS trait in both populations. Likewise, for NPB, the number of branches produced by many of the RILs was higher than the parental lines in both populations, which indicated a positive transgressive segregation for this trait, although some skewing was observed in MA to low values. For LI, the histograms showed a similar pattern across both populations, indicating positive transgressive segregation. Hence, in general, the phenotypic segregations for rate of production traits in these two RIL populations exhibited normal distribution, and transgressive segregation in both directions, a typical phenomenon of a quantitative trait, regulated by several genes and influenced by the environment.

Mean values, standard errors, and ranges of variation for the quantitative traits in each RIL mapping population for each environmental condition have been summarized in Supplementary Tables 2, 3. Mesoamerican PHA0419 and PHA0399 lines were late in flowering and taller, with larger rates of plant production compared to Beluga. In both RIL populations and for all evaluated traits, it was found differences among environments in mean values and ranges of variation, although environment × line interactions were not significant for most of the evaluated traits in both RIL populations. Significant differences among parents were detected except in some environments for PST, LL, LWI, PL, and PWI traits in the MA RIL population (Supplementary Table 2) and for BL, PWI, and ST traits in the AM RIL population (Supplementary Table 3). Despite of that, it was observed significant differences among RILs for all quantitative traits except for LL in MA population under F108 and F109 environmental conditions (Supplementary Table 2).

High broad-sense heritability estimates (≥ 0.50; Supplementary Table 4) were detected for most of the quantitative traits across the four environments except for NPB, LI and BWI in both populations, and PST, BL, and PT in MA population. Higher heritability estimates and correlations were observed in AM population than in MA population. LMS and FT were significantly correlated in both AM (*r* = 0.50, *P* ≤ 0.001) and MA (*r* = 0.38, *P* ≤ 0.001) populations. Their inter-relationship suggests that some genomic regions influence both traits. Finally, a negative and significant correlation was observed between FT and NPB (*r* = –0.43, *P* ≤ 0.001) and positive and significant correlation between FT and LI (*r* = 0.33, *P* ≤ 0.001) in AM population, while no significant correlation values were observed in MA population. These correlations indicated that, on average, genotypes with a low number of primary branches, and high length of internodes showed a later flowering date and a longer main stem. As expected from the ontogenetic pattern of vegetative growth, LMS-values were positive and significant correlated with plant parts size (bracteole, leaf, pod, and seed) in both populations except for BWI in AM RIL, suggesting a pleiotropic effect. Positive and significant correlations were observed between FT, PGT, and PST; these last traits were positively correlated with LI in both RIL populations. No significant or low correlation values were observed between FT and all other rate of plant production traits measured. Significant and positive correlations were found between PST and rate of plant production traits. In contrast, the correlations with PGT were negative. SY presented a significant and low positive correlation value with FT in the MA and AM RILs (*r* = 0.16, *r* = 0.15, *P* ≤ 0.01). The correlations between SY and PGT, and PST were not significant except for PST in the MA RIL (*r* = 0.37, *P* ≤ 0.001).

### Marker segregation analysis and consensus genetic map construction

Six hundred and thirty-four markers were screened for DNA polymorphisms in the parents of MA RIL, which rendered a 36% polymorphism rate. Sixty-two (27%) out of the 228 polymorphic markers evaluated in the MA RIL population exhibited segregation distorsion and thus, they could not be mapped. Finally, the genetic map of the MA RIL population (Figure not shown) consisted of 166 loci (158 SSRs, 1 SCAR, 6 SNPs and the *FIN* locus) distributed in 11 LGs. Out of 166 markers, 56 and 110 were dominant and codominant, respectively. LGs were named as reported in Pedrosa-Harand et al. (2008) using for the assignment of LG number and orientation 55 common SSR markers previously mapped (Freyre et al., 1998; Blair et al., 2008, 2010, 2011; Cichy et al., 2009; García et al., 2011). The map covered a genetic distance of 1188.9 cM, with an average of 7.2 cM among markers, which ranged from 5.3 cM (Pv02) to 16.0 cM (Pv05). The average genetic distance per LG was 108.1 cM, ranging from 70.6 cM (Pv10) to 134.1 cM (Pv07). A detailed description of this map is provided in Supplementary Table 5.

Likewise, 364 markers were screened for DNA polymorphisms in the parents of AM RIL, which rendered a 39% polymorphism rate. A total of 245 polymorphic markers were evaluated in the AM RIL population. Sixty-five (26%) out of these 245 markers, could not be mapped as they were not linked to other markers. Thus, the AM genetic map was constructed with a total of 180 loci (179 SSRs and *FIN* locus), from which 73 were dominant and 107 codominant. These loci were distributed among 11 LGs (Figure not shown) that covered a genetic distance of 1175.5 cM. The density of markers ranged from 3.4 cM (Pv01) to 12.6 cM (Pv04), with an average of 6.9 cM per LG. The longest LG was Pv08 (144.4 cM), whereas Pv11 was the shortest LG (48.2 cM), with an average genetic distance of 106.9 cM per LG. A complete description of the AM map is shown in Supplementary Table 6.

**Figure 2:**
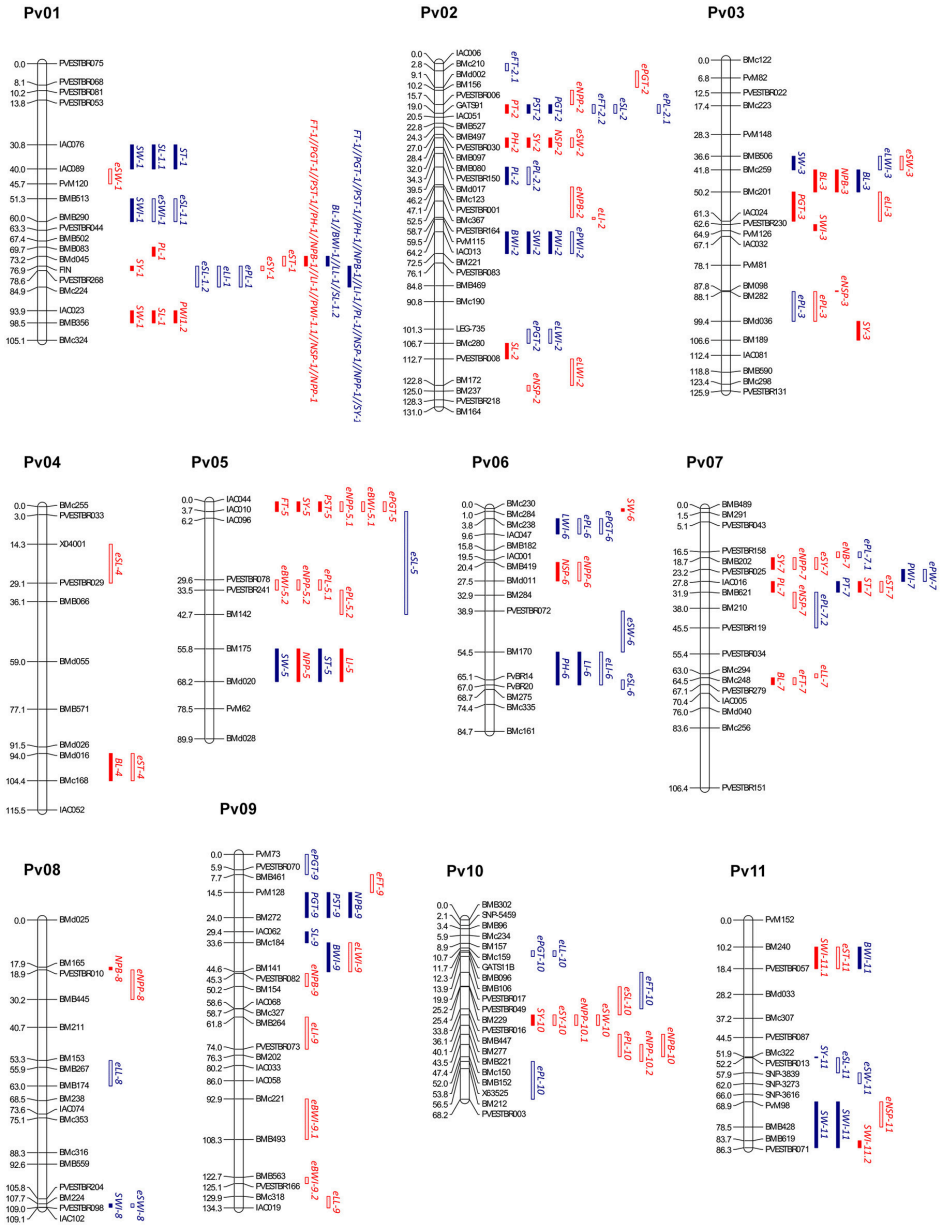
**Common bean consensus map showing the location of main-effect QTLs and E-QTLs for quantitative traits from the MA (blue color) and AM (red color) RIL populations**. Distances among markers are indicated in cM to the left of the LGs. Names of markers are shown on the right. QTLs are depicted as vertical bars to the right of the LG. Names of QTLs are listed in Tables 2–5. Main effect QTLs are indicated with solid bars and E-QTLs are indicated with hatched bars.

The construction of a consensus map (Figure 2) was performed by connecting the MA and AM mapping data. To integrate both MA and AM maps in a single consensus map, 103 common markers were used as anchor points. As a result, marker segregation data were assembled for a total of 202 marker loci (196 SSRs, 5 SNPs, and the *FIN* locus) into 11 LGs. The total length of the consensus genetic map was 1156.2 cM and had a marker average density of one marker per 6.1 cM (Table 1). The marker order of the integrated map was largely collinear with the two individual maps, although a few local inversions and marker rearrangements over short intervals were observed. Most of the markers from both RIL populations showed a good linear relationship between their position on the genetic map and on the physical map of the common bean genome (Supplementary Table 7).

**Table 1:** **Consensus map constructed from MA and AM RIL maps**.

**Linkage groups**	**Map length (cM)**	**No. of markers**	**Marker density (cM/marker)**	**Marker types**		
				**SSR**	**SNP**	***FIN***
1	105.1	19	5.5	18	–	1
2	131	31	4.2	30	1	–
3	125.8	21	6	21	–	–
4	115.4	11	10.5	11	–	–
5	89.9	10	9	10	–	–
6	84.7	16	5.3	16	–	–
7	106.4	18	5.9	18	–	–
8	109.1	17	6.4	17	–	–
9	134.3	23	5.8	23	–	–
10	68.2	21	3.2	20	1	–
11	86.3	15	5.7	12	3	–
Total	1156.2	202	6.1	196	5	1

### Multiple environment single-locus QTL analysis

QTL analysis based on MCIM mapping using QTLNetwork 2.0 was undertaken to identify single-locus QTLs across all environments. The positions of QTLs and their confidence intervals for the traits on the consensus map are shown in Figure 2 and Tables 2, 3. Thus, multi-environment QTL analyses allowed for the detection of 43 and 40 single-locus QTLs with significant additive main effects and/or QE effects for the evaluated quantitative traits in MA and AM RILs, respectively.

**Table 2:** **Single-locus QTLs and QTLs × Environment (QE) interaction effects identified in the MA RIL population grown in four different environments**.

**QTL**	**Marker interval**	**LG (pos.)^a^**	***F*-value^b^**	**A^c^**	***R*^2^(a)^d^**	**QE AE^e^**			***R*^2^(ae)^f^**
**FLOWERING AND MATURITY TRAITS**									
*FT-1^*MA*^*	*FIN-*BMC224	Pv01 (76.9–84.9)	17.33	1.43^**^	18.96		ns		
*PGT-1^*MA*^*	*FIN-*BMC224	Pv01 (76.9–84.9)	21.26	1.53^**^	10.26	−1.34	^**^	F108	2.79
						1.20	^**^	G108	1.90
						0.86	^**^	G109	0.95
*PGT-2^*MA*^*	PVESTBR006-GATs91	Pv02 (15.7–19.0)	8.28	0.91^**^	4.24	−0.72	^*^	F108	0.78
						0.81	^*^	G108	0.64
						0.94	^**^	G109	0.87
						−1.01	^*^	F109	1.04
*PGT-9^*MA*^*	PVM128-BM272	Pv09 (14.5–24.0)	7.42	1.16^**^	3.84	1.1	^**^	G109	1.14
*PST-1^*MA*^*	*FIN-*BMC224	Pv01 (76.9–84.9)	12.73	1.66^**^	7.62	0.91	^**^	G108	1.39
*PST -2^*MA*^*	PVESTBR006-GATs91	Pv02 (15.7–19.0)	8.45	2.08^**^	6.69		ns		
*PST -9^*MA*^*	PVM128-BM272	Pv09 (14.5–24.0)	6.75	1.48^**^	4.29	1.04	^*^	G109	1.33
**VEGETATIVE GROWTH TRAITS**									
*LMS-1^*MA*^*	*FIN-*BMC224	Pv01 (76.9–84.9)	181.35	0.56^**^	66.08		ns		
*LMS-6^*MA*^*	BM170-PVBR20	Pv06 (54.5–66.9)	9.23	0.12^**^	5.56		ns		
*NPB-1^*MA*^*	*FIN-*BMC224	Pv01 (76.9–84.9)	12.73	−0.13^**^	7.79		ns		
*NPB-9^*MA*^*	PVM128-BM272	Pv09 (14.5–24.0)	9.17	0.11^**^	6.27		ns		
*LI-1^*MA*^*	*FIN-*BMC224	Pv01 (76.9–84.9)	12.05	0.04^**^	16.41	0.13	^**^	G108	6.37
						−0.06	^*^	F109	1.42
*LI-6^*MA*^*	BM170-PVBR20	Pv06 (54.5–66.9)	7.79	0.05^**^	3.29	−0.05	^*^	F108	1.09
						0.08	^**^	G108	3.12
**PLANT PRODUCTION TRAITS**									
*BL-1^*MA*^*	BMD045-*FIN*	Pv01 (73.2–76.9)	7.22	0.03^**^	3.09	0.05	^**^	G108	2.12
						−0.04	^**^	F109	1.86
*BL-3^*MA*^*	BMC259-BMC201	Pv03 (41.8–50.2)	7.8	−0.02^**^	13.17	−0.03	^**^	G108	1.33
						0.03	^*^	F109	1.64
*BWI-1^*MA*^*	BMD045-*FIN*	Pv01 (73.2–76.9)	7.05	0.03^**^	3.06		ns		
*BWI-2^*MA*^*	IAC013-BM221	Pv02 (64.2–72.5)	6.93	−0.04^**^	4.02		ns		
*BWI-9^*MA*^*	BMC184-BM141	Pv09 (33.6–44.6)	7.38	−0.02^**^	1.77		ns		
*BWI-11^*MA*^*	BM240-PVESTBR057	Pv11(10.2–18.4)	7.17	−0.03^**^	3.31		ns		
*LL-1.1^*MA*^*	BMD045-*FIN*	Pv01 (73.2–76.9)	7.05	0.01^**^	2.85	0.02	^**^	G108	2.71
						−0.01	^**^	G109	1.51
						−0.01	^**^	F109	1.54
*LWI-6^*MA*^*	BMc238-IAC047	Pv06 (3.8–9.6)	6.73	−0.16^**^	3.70		ns		
*PL-1^*MA*^*	*FIN-*BMC224	Pv01 (76.9–84.9)	7.07	0.39^**^	7.59		ns		
*PL-2^*MA*^*	BMD017-BMC123	Pv02 (39.5–46.2)	10.81	−0.40^**^	4.75		ns		
*PWI-2^*MA*^*	IAC013-BM221	Pv02 (64.2–72.5)	7.1	−0.24^**^	2.56	0.21	^*^	F108	1.86
*PWI-7^*MA*^*	PVESTBR025-IAC016	Pv07 (23.2–27.8)	10.85	−0.34^**^	3.68	0.26	^*^	F108	1.81
*PT-7^*MA*^*	IAC016- BMB621	Pv07 (27.8–31.9)	7.22	−0.20^**^	2.88	0.25	^*^	F108	2.07
*SL-1.1^*MA*^*	IAC076-IAC089	Pv01 (30.8–40.0)	7.86	0.34^**^	4.96		ns		
*SL-1.2^*MA*^*	BMD045-*FIN*	Pv01 (73.2–76.9)	10.09	0.40^**^	4.82		ns		
*SL-9^*MA*^*	IAC062-BMC184	Pv09 (29.4–33.6)	7.91	−0.44^*^	9.65		ns		
*SWI-1^*MA*^*	BMB513-BMB290	Pv01 (51.3–60.0)	7.85	0.26^**^	5.23		ns		
*SWI-2^*MA*^*	IAC013-BM221	Pv02 (64.2–72.5)	7.22	−0.16^**^	3.47	0.11	^*^	F108	0.51
*SWI-8^*MA*^*	BM224-PVESTBR098	Pv08 (107.6–109.0)	8.11	0.24^**^	3.03		ns		
*SWI-11^*MA*^*	PVM98-PVESTBR071	Pv11 (68.9–86.3)	7.93	−0.20^**^	3.34		ns		
*ST-1^*MA*^*	IAC076-IAC089	Pv01 (30.8–40.0)	6.96	0.09^**^	1.92		ns		
*ST-5^*MA*^*	BM175-BMD020	Pv05 (55.8–68.2)	6.7	−0.13^**^	4.40		ns		
*SW-1^*MA*^*	IAC076-IAC089	Pv01 (30.8–40.0)	6	0.06^**^	4.87		ns		
*SW-3^*MA*^*	BMB506-BMC259	Pv03 (36.6–41.8)	9.21	0.07^**^	4.38		ns		
*SW-5^*MA*^*	BM175-BMD020	Pv05 (55.8–68.2)	6.61	−0.05^**^	5.12		ns		
*SW11^*MA*^*	PVM98-PVESTBR071	Pv11 (68.9–86.3)	6.29	−0.05^**^	4.81		ns		
*NSP-1^*MA*^*	*FIN-*BMC224	Pv01 (76.9–84.9)	13.21	0.24^**^	7.90	−0.15	^**^	F108	1.65
						0.18	^**^	G108	2.19
*NPP-1^*MA*^*	*FIN-*BMC224	Pv01 (76.9–84.9)	17.76	0.48^**^	9.51	−0.29	^**^	F108	2.17
						0.36	^**^	G108	1.92
						0.2	^*^	G109	0.76
*SY-1^*MA*^*	*FIN-*BMC224	Pv01 (76.9–84.9)	32.15	0.37^**^	18.90	−0.17	^**^	F108	1.91
						0.12	^*^	G108	0.84
*SY-11^*MA*^*	BMC322-PVESTBR013	Pv11 (51.9–52.2)	8.37	0.23^**^	6.12		ns		

a *Linkage group and the estimated confidence interval of QTL position in the consensus map (in Kosambi cM)*.

b *F-values of significance of each QTL*.

c *Estimated additive effect. Positive values indicate that alleles from PHA0419 have a positive effect on the traits, and negative values indicate that positive effect on the traits is due to the presence of the alleles from BELUGA*.

d *Percentage of the phenotypic variation explained by additive effects*.

e *Predicted additive by environment interaction effect. The meaning of sign values is described in the second footnote (^c^)*.

f *Percentage of the phenotypic variation explained by additive by environment interaction effect*.

* *P ≤ 0.05*,

** *P ≤ 0.01. Experiment wide P-value. Only significant effects are listed. ns, not significant effects on the four environments evaluated*.

*FT, flowering time; PGT, pod green time; PST, physiological maturity time; LMS, length of main stem; NPB, the number of primary stem branches; LI, internode length; BL, bracteole length; BWI, bracteole width; LL, leaflet length; LWI, leaflet width; PL, pod length; PWI, pod width; PT, pod thickness; SL, seed length; SWI, seed width; ST, seed thickness; SW, 100 seed weight; NSP, number of seeds per pod; NPP, number of pods per plant; SY, seed yield*.

**Table 3:** **Single-locus QTLs and QTLs × Environment (QE) interaction effects identified in the AM RIL population grown in four different environments**.

**QTL**	**Marker interval**	**LG (pos.)^a^**	***F*-value^b^**	**A^c^**	***R*^2^(a)^d^**	**QE AE^e^**			***R*^2^(ae)^f^**
**FLOWERING AND MATURITY TRAITS**									
*FT-1^*AM*^*	BMD045-*FIN*	Pv01 (73.2–76.9)	21.37	−6.82^**^	30.74		ns		
*FT-5^*AM*^*	IAC044-IAC010	Pv05 (0.0–3.7)	5.91	−1.99^**^	10.70		ns		
*PGT-1^*AM*^*	BMD045-*FIN*	Pv01 (73.2–76.9)	16.49	−5.54^**^	21.29	−4.59	^**^	G108	4.96
*PGT-3^*AM*^*	BMC201-IAC024	Pv03 (50.2–61.3)	5.4	1.61^**^	3.82	2.13	^**^	F109	1.96
*PST-1^*AM*^*	BMD045-*FIN*	Pv01(73.2–76.9)	25.07	−8.94^**^	31.91	6.06	^*^	F109	1.43
						−15.2	^**^	G108	18.89
*PST-5^*AM*^*	IAC044-IAC010	Pv05 (0.0–3.7)	6.27	−2.29^**^	9.13		ns		
**VEGETATIVE GROWTH TRAITS**									
*LMS-1^*AM*^*	BMD045-*FIN*	Pv01 (73.2–76.9)	33.98	−170.17^**^	50.27	97.4	^**^	F109	9.51
*LMS-2^*AM*^*	BMB097-BMB080	Pv02 (28.4–32.0)	8.8	55.33^**^	5.97	76.8	^**^	F109	4.25
*NPB-1^*AM*^*	BMD045-*FIN*	Pv01 (73.2–76.9)	14.85	0.15^**^	9.23		ns		
*NPB-3^*AM*^*	BMC259-BMC201	Pv03 (41.8–50.2)	8.97	−0.02^*^	2.95	0.08	^*^	F109	3.11
*NPB-8^*AM*^*	BM165-PVESTBR010	Pv08 (17.8–18.8)	10.75	−0.05^*^	5.29		ns		
*LI-1^*AM*^*	BMD045-*FIN*	Pv01 (73.2–76.9)	107.31	−4.46^**^	34.28	4.4	^**^	F108	9.24
						2.43	^**^	F109	2.57
						−4.07	^**^	G108	6.6
						−6.78	^**^	G109	9.84
*LI-5^*AM*^*	BM175-BMD020	Pv05 (55.8–68.2)	5.4	0.99^**^	1.55		ns		
**PLANT PRODUCTION TRAITS**									
*BL-3^*AM*^*	BMC259-BMC201	Pv03 (41.8–50.2)	9.28	0.10^**^	17.32		ns		
*BL-4^*AM*^*	BMD016-BMC168	Pv04 (94.0–104.4)	5.77	0.09^**^	2.45		ns		
*BL-7^*AM*^*	BMC248-PVESTBR279	Pv07 (64.5–67.1)	8.17	−0.04^**^	7.15		ns		
*PL-1^*AM*^*	BMB083-BMD045	Pv01 (69.7–73.2)	5.8	0.24^**^	3.98	−0.18	^**^	F108	3.85
*PL-7^*AM*^*	IAC016-BMB621	Pv07 (27.8–31.9)	8.73	0.56^**^	10.37	−0.15	^**^	F108	3.66
*PWI-1.1^*AM*^*	BMD045-*FIN*	Pv01 (73.2–76.9)	7.11	−0.65^**^	8.74	0.53	^*^	F108	2.91
*PWI-1.2^*AM*^*	IAC023-BMB356	Pv01 (93.9–98.5)	6.62	0.83^**^	6.55		ns		
*PT-2^*AM*^*	PVESTBR006-GATS91	Pv02 (15.7–19.0)	6.36	0.25^**^	5.60		ns		
*SL-1^*AM*^*	IAC023-BMB356	Pv01 (93.9–98.5)	9.05	1.88^**^	24.75		ns		
*SL-2^*AM*^*	BMC280-PVESTBR008	Pv02 (106.7–112.7)	5.1	−0.23^**^	5.65		ns		
*SWI-3^*AM*^*	PVESTBR230-BMB339	Pv03 (62.5–64.9)	10.36	−0.27^**^	10.95		ns		
*SWI-11.1^*AM*^*	BM240-PVESTBR057	Pv11 (10.2–18.4)	9.1	−0.22^**^	13.29		ns		
*SWI-11.2^*AM*^*	BMB619-PVESTBR071	Pv11 (83.7–86.3)	8.32	−0.49^**^	17.64		ns		
*ST-7^*AM*^*	IAC016-BMB621	Pv07 (27.8–31.9)	5.89	0.18^**^	11.08		ns		
*SW-1^*AM*^*	IAC023-BMB356	Pv01 (93.9–98.5)	5.98	0.13^**^	10.91		ns		
*SW-6^*AM*^*	BMC230-BMC284	Pv06 (0.0–1.0)	5.59	0.04^**^	3.80		ns		
*NSP-1^*AM*^*	BMD045-*FIN*	Pv01 (73.2–76.9)	14.76	−0.46^**^	14.01	0.31	^**^	F108	2.09
						−0.62	^**^	F109	9.65
*NSP-2^*AM*^*	BMB097-BMB080	Pv02 (28.4–32.0)	6.06	0.32^**^	6.39	0.3	^**^	F109	3.24
*NSP-6^*AM*^*	BMB419-BMD011	Pv06 (20.4–27.5)	6.96	−0.37^**^	5.91	0.24	^*^	F108	1.15
						−0.54	^**^	F109	6.79
*NPP-1^*AM*^*	BMD045-*FIN*	Pv01 (73.2–76.9)	18.04	−0.82^**^	10.14	0.69	^**^	F108	2.73
						−1.64	^**^	F109	14.71
*NPP-5^*AM*^*	BM175-BMD020	Pv05 (55.8–68.2)	6.20	0.42^**^	4.10	−0.64	^**^	F109	4.37
*SY-1^*AM*^*	*FIN*-PVESTBR268	Pv01 (76.9–78.6)	9.17	−8.44^**^	14.75	−7.72	^**^	F109	6.55
*SY-2^*AM*^*	BMB097-BMB080	Pv02 (28.4–32.0)	9.26	6.03^**^	1.32	6.06	^**^	F108	0.10
						9.17	^**^	F109	2.42
						5.97	^**^	G108	0.14
*SY-3^*AM*^*	BMD036-BM189	Pv03 (99.4–106.6)	5.65	−2.09^**^	2.66	2.09	^*^	G108	0.68
*SY-5^*AM*^*	IAC044-IAC010	Pv05 (0.0–3.7)	5.71	0.86^*^	4.48	−5.73	^**^	F109	3.57
*SY-7^*AM*^*	BMB202-PVESTBR025	Pv07 (18.7–23.2)	6.12	−3.59^**^	7.10	3.34	^**^	F108	0.39
						3.44	^**^	G108	0.85
*SY-10^*AM*^*	BMB447-BM277	Pv10 (36.1–40.1)	10.31	0.20^*^	1.80	4.05	^**^	F109	1.30

a *Linkage group and the estimated confidence interval of QTL position in the consensus map (in Kosambi cM)*.

b *F-values of significance of each QTL*.

c *Estimated additive effect. Positive values indicate that alleles from BELUGA have a positive effect on the traits, and negative values indicate that positive effect on the traits is due to the presence of the alleles from PHA0399*.

d *Percentage of the phenotypic variation explained by additive effects*.

e *Predicted additive by environment interaction effect. The meaning of sign values is described in the second footnote (^c^)*.

f *Percentage of the phenotypic variation explained by additive by environment interaction effect*.

* *P ≤ 0.05*,

** *P ≤ 0.01. Experiment wide P-value. Only significant effects are listed. ns, not significant effects on the four environments evaluated*.

*FT, flowering time; PGT, pod green time; PST, physiological maturity time; LMS, length of main stem; NPB, the number of primary stem branches; LI, internode length; BL, bracteole length; BWI, bracteole width; LL, leaflet length; LWI, leaflet width; PL, pod length; PWI, pod width; PT, pod thickness; SL, seed length; SWI, seed width; ST, seed thickness; SW, 100 seed weight; NSP, number of seeds per pod; NPP, number of pods per plant; SY, seed yield*.

The distribution of the flowering and maturity time QTLs in the MA RIL population (Table 2) varied from 3 on Pv01 (*FT-1*^*MA*^, *PGT-1*^*MA*^, *PST-1*^*MA*^), to 2 on each Pv02 (*PGT-2*^*MA*^, *PST-2*^*MA*^), and Pv09 (*PGT-9*^*MA*^, *PST-9*^*MA*^). All these QTLs had a positive additive value, indicating that alleles from the PHA0419 parent increase flowering and maturity times, whose main additive effects accounting for 3.84 (*PGT-9*^*MA*^) to 18.96% (*FT-1*^*MA*^) of the phenotypic variance for these traits. Five out of the seven QTLs identified showed QE interactions effects, ranged from 0.64 (for *PGT-2*^*MA*^ in G108) to 2.79% (for *PGT-1*^*MA*^ in F108). In addition, for the AM RIL population (Table 3), six single-locus QTLs were detected, three on Pv01 (*FT-1*^*AM*^, *PGT-1*^*AM*^, *PST-1*^*AM*^), two on Pv05 (*FT-5*^*AM*^, *PST-5*^*AM*^), and one on Pv03 (*PGT-3*^*AM*^). The additive effects of these QTLs explained a phenotypic variance up to 30.74% (*FT-1*^*AM*^). Three of them exhibited significant QE interaction effects, which varied from 1.43 (for *PST-1*^*AM*^ in F109) to 18.89% (for *PST-1*^*AM*^ in G108). Most of the QTLs had a negative additive value, except for the QTL *PGT-3*^*AM*^, which indicated that alleles from the PHA0399 parent mainly enhance flowering and maturity times. Combining both QTL mapping results, two genomic regions stood out, BMD045-*FIN* and *FIN*-BMC224 in MA and AM RIL populations, respectively, which contained QTLs controlling FT, PGT, and PST traits that were correlated in both mapping populations (Supplementary Table 4).

For vegetative growth habit traits, six single-locus QTLs were detected in the MA RIL population (Table 2), three located on Pv01 (*LMS-1*^*MA*^, *NPB-1*^*MA*^, *LI-1*^*MA*^), two on Pv06 (*LMS-6*^*MA*^, *LI-6*^*MA*^), and one on Pv09 (*NPB-9*^*MA*^), whose additive effects explained a total phenotypic variance that ranged from 3.29 (*LI-6*^*MA*^) to 66.08% (*LMS-1*^*MA*^). Two out of the six QTLs showed QE interaction effects, which ranged from 1.09 to 6.37% (for *LI-6*^*MA*^ in F108 and for *LI-1*^*MA*^ in G108, respectively). Regarding the AM RIL population, seven single-locus QTLs were identified (Table 3), three on Pv01 (*LMS-1*^*AM*^, *NPB-1*^*AM*^, *LI-1*^*AM*^), and one on each Pv02 (*LMS-2*^*AM*^), Pv03 (*NPB-3*^*AM*^), Pv05 (*LI-5*^*AM*^), and Pv08 (*NPB-8*^*AM*^). The additive effects of these QTLs accounting for 1.55 (*LI-5*^*AM*^) to 50.27% (*LMS-1*^*AM*^); and four of them showed QE interaction effects that ranged from 2.57 to 9.84% (for *LI-1*^*AM*^ in F109 and G109, respectively). In both RIL populations, the same genomic region on Pv01 (*FIN*-BMC224 and BMD045-*FIN*) contained QTLs for LMS, NPB, and LI traits. On this region, Mesoamerican alleles contributed to enhance the LMS and LI traits, whereas Andean alleles increased the NPB trait (Tables 2, 3).

Regarding plant production traits, 30 single-locus QTLs were detected in the MA RIL population (Table 2), 12 located on Pv01 (*BL-1*^*MA*^, *BWI-1*^*MA*^, *LL-1.1*^*MA*^, *PL-1*^*MA*^, *SL-1.1*^*MA*^, *SL-1.2*^*MA*^, *SWI-1*^*MA*^, *ST-1*^*MA*^, *SW-1*^*MA*^, *NSP-1*^*MA*^, *NPP-1*^*MA*^, *SY-1*^*MA*^), four on each Pv02 (*BWI-2*^*MA*^, *PL-2*^*MA*^, *PWI-2*^*MA*^, *SWI-2*^*MA*^) and Pv11 (*BWI-11*^*MA*^, *SWI-11*^*MA*^, *SW11*^*MA*^, *SY-11*^*MA*^), two on each Pv03 (*BL-3*^*MA*^, *SW-3*^*MA*^), Pv05 (*ST-5*^*MA*^, *SW-5*^*MA*^), Pv07 (*PWI-7*^*MA*^, *PT-7*^*MA*^), and Pv09 (*BWI-9*^*MA*^, *SL-9*^*MA*^), as well as one on each Pv06 (*LWI-6*^*MA*^) and Pv08 (*SWI-8*^*MA*^). Their additive effects accounting for a phenotypic variance that ranged from 1.77 (*BWI-9*^*MA*^) to 18.90% (*SY-1*^*MA*^). Ten out of the 30 single-locus QTLs displayed environment interactions effects that ranged from 0.51 to 2.71% (for *SWI-2*^*MA*^ and *LL-1.1*^*MA*^ in F108 and G108, respectively). Additionally, for the AM RIL population (Table 3), 27 single-locus QTLs were identified, eight located on Pv01 (*PL-1*^*AM*^, *PWI-1.1*^*AM*^, *PWI-1.2*^*AM*^, *SL-1*^*AM*^, *SW-1*^*AM*^, *NSP-1*^*AM*^, *NPP-1*^*AM*^, *SY-1*^*AM*^), four on each Pv02 (*PT-2*^*AM*^, *SL-2*^*AM*^, *NSP-2*^*AM*^, *SY-2*^*AM*^) and Pv07 (*BL-7*^*AM*^, *PL-7*^*AM*^, *ST-7*^*AM*^, *SY-7*^*AM*^), three on Pv03 (*BL-3*^*AM*^, *SWI-3*^*AM*^, *SY-3*^*AM*^), two on each Pv05 (*NPP-5*^*AM*^, *SY-5*^*AM*^), Pv06 (*SW-6*^*AM*^, *NSP-6*^*AM*^), and Pv11 (*SWI-11.1*^*AM*^, *SWI-11.2*^*AM*^), and one on each Pv04 (*BL-4*^*AM*^) and Pv10 (*SY-10*^*AM*^). The additive effects of these QTLs accounting for 1.32 (*SY-2*^*AM*^) to 24.75% (*SL-1*^*AM*^). Fourteen out of the 27 single-locus QTLs displayed environment interactions, whose effects explaining a phenotypic variance up to 14.71% (*NPP-1*^*AM*^ in F109). Furthermore, in MA and AM RIL populations, positive, and negative additive values were identified, indicating that alleles from both Mesoamerican and Andean parents have a positive agronomical effect on the crop growth and productivity traits.

### Epistatic QTL interactions

Epistatic and environment interactions among QTLs were detected by means of a two-dimensional genome scan using QTLNetwork 2.0. Thus, a total of 33 and 49 significant E-QTLs involved in 17 and 25 epistatic interactions were identified for the MA and AM RIL populations, respectively (Tables 4, 5). Most of these interactions were due to loci without detectable QTL additive main-effects, and only five and three E-QTLs had both individual additive and epistatic effects in the MA and AM RIL populations, respectively. No significant epistastic interactions were detected for PST, LMS, BL, and PT traits in both RIL populations; whereas E-QTLs were not identified for NPB, ST, NSP, NPP, and SY, and for PWI and SWI traits in the MA and AM RIL population, respectively (Tables 4, 5).

**Table 4:** **Epistatic QTLs (E-QTLs) and E-QTL × Environment (E-QE) interaction effects detected in the MA RIL populations grown in four different environments**.

***e-QTLi*^a^**	**Marker interval**	**LG (pos.)^b^**	***e-QTLj*^a^**	**Marker interval**	**LG (pos.)**	***F*-value^c^**	**AA^d^**	***R*^2^(aa)^e^**	**e-QE AAE^f^**			***R*^2^ (aae)^g^**
**FLOWERING AND MATURITY TRAITS**												
*eFT-2.1^*MA*^*	IAC006-BMC210	Pv02 (0.00–2.8)	*eFT-10^*MA*^*	PVESTBR017-PVESTBR016	Pv10 (19.9–33.8)	11.88	1.07^**^	3.82	1.27	^**^	G109	1.10
*eFT-2.2^*MA*^*	PVESTBR006-GATS91	Pv02 (15.7–19.0)	*eFT-10^*MA*^*	PVESTBR017-PVESTBR016	Pv10 (19.9–33.8)	9.59	0.92^**^	2.47		ns		
*ePGT-2^*MA*^*	LEG-735-BMc280	Pv02 (101.3–106.4)	*ePGT-10^*MA*^*	GATS11B-BMB106	Pv10 (11.7–13.9)	5.82	−1.56^**^	1.09	1.98	^*^	F108	1.02
									−1.75	^*^	G109	0.72
*ePGT-6^*MA*^*	BMc238-IAC047	Pv06 (3.8–9.6)	*ePGT-9^*MA*^*	PVM73-BMB461	Pv09 (0.0–7.6)	5.61	−0.81^**^	1.61		ns		
**VEGETATIVE GROWTH TRAITS**												
*eLI-1^*MA*^*	*FIN-*BMC224	Pv01 (76.9–84.9)	*eLI-6^*MA*^*	BM170-PVBR20	Pv06 (54.5–67.0)	5.89	−0.06^**^	2.34		ns		
**PLANT PRODUCTION TRAITS**												
*eLL-8^*MA*^*	BM153-BMB174	Pv08 (53.3–63.0)	*eLL-10^*MA*^*	GATS11B-BMB106	Pv10 (11.7–13.9)	7.89	−0.01^**^	3.69		ns		
*eLWI-2^*MA*^*	LEG-735-BMC280	Pv02 (101.3–106.7)	*eLWI-3^*MA*^*	BMB506-BMC259	Pv03 (36.6–41.8)	6.82	−0.27^**^	6.03	−0.13	^*^	G108	1.09
*ePL-1^*MA*^*	*FIN-*BMC224	Pv01 (76.9–84.9)	*ePL-2.2^*MA*^*	BMD017-BMC123	Pv02 (39.5–46.2)	5.75	−0.19^*^	1.14	0.26	^*^	F108	1.06
*ePL-2.1^*MA*^*	PVESTBR006-GATS91	Pv02 (15.7–18.9)	*ePL-10^*MA*^*	X63525-PVESTBR003	Pv10 (53.8–68.2)	9.61	−0.38^**^	3.70		ns		
*ePL-3^*MA*^*	BM282-BMD036	Pv03 (88.1–99.4)	*ePL-7.1^*MA*^*	PVESTBR158-BMB202	Pv07 (16.5–18.7)	11.77	−0.58^**^	4.08		ns		
*ePL-6^*MA*^*	BMC238-IAC047	Pv06 (3.8–9.6)	*ePL-7.2^*MA*^*	BMB621-PVESTBR119	Pv07 (31.9–45.5)	9.65	0.41^**^	3.62		ns		
*ePWI-2^*MA*^*	IAC013-BM221	Pv02 (64.2–72.5)	*ePW-7^*MA*^*	PVESTBR025-IAC016	Pv07 (23.2–27.8)	8.14	0.21^**^	1.11		ns		
*eSL-1.1^*MA*^*	BMB513-BMB290	Pv01 (51.3–59.9)	*eSL-2^*MA*^*	PVESTBR006- GATS91	Pv02 (15.7–18.9)	7.34	−0.86^**^	8.30		ns		
*eSL-1.2^*MA*^*	*FIN-*BMC224	Pv01 (76.9–84.9)	*eSL-11^*MA*^*	PVESTBR013-SNP-3839	Pv11 (52.2–57.9)	7.3	−0.27^**^	1.51		ns		
*eSL-5^*MA*^*	IAC010-BM142	Pv05 (3.7–42.7)	*eSL-6^*MA*^*	PVBR14-BM275	Pv06 (65.1–68.7)	7.83	−0.57^**^	3.89	0.62	^**^	F108	1.07
*eSWI-1^*MA*^*	BMB513-BMB290	Pv01 (51.3–59.9)	*eSWI-8^*MA*^*	BM224-PVESTBR098	Pv08 (107.6–109.0)	6.64	0.23^**^	1.91		ns		
*eSW-6^*MA*^*	PVESTBR072-BM170	Pv06 (38.9–54.5)	*eSW-11^*MA*^*	SNP-3839-SNP-3273	Pv11 (57.9–62.0)	7.87	0.05^**^	2.83	−0.09	^**^	F109	1.29

a *E-QTLi and E-QTLj are the two QTLs involved in epistatic interaction*.

b *Linkage group and the estimated confidence interval of QTL position in the consensus map (in Kosambi cM)*.

c *F-values of significance of each QTL*.

d *Estimated additive by additive epistatic effect. Positive values indicate that alleles from PHA0419 have a positive effect on the traits, and negative values indicate that positive effect on the traits is due to the presence of the alleles from BELUGA*.

e *Percentage of the phenotypic variation explained by additive by additive epistatic effects*.

f *Predicted additive by additive epistatic effect by environment interaction effect. The meaning of sign values is described in the third footnote (^d^)*.

g *Percentage of the phenotypic variation explained by additive by additive epistatic effect by environment interaction effect*.

* *P ≤ 0.05*,

** *P ≤ 0.01. Experiment wide P-value. Only significant effects are listed. ns, not significant effects on the four environments evaluated*.

*FT: flowering time; PGT: pod green time; LI: internode length; LL: leaflet length; LWI: leaflet width; PL: pod length; PWI: pod width; SL: seed length; SWI: seed width; SW: 100 seed weight*.

**Table 5:** **Epistatic QTLs (E-QTLs) and E-QTL × Environment (E-QE) interaction effects detected in the AM RIL populations grown in four different environments**.

***e-QTLi*^a^**	**Marker interval**	**LG (pos.)^b^**	***e-QTLj*^a^**	**Marker interval**	**LG (pos.)**	***F*-value^c^**	**AA^d^**	***R*^2^(aa)^e^**	**e-QE AAE^f^**			***R*^2^ (aae)^g^**
**FLOWERING AND MATURITY TRAITS**												
*eFT-7^*AM*^*	BMC248-PVESTBR279	Pv07 (64.5–67.1)	*eFT-9^*AM*^*	BMB461-PVM128	Pv09 (7.6–14.5)	8.08	−6.03^**^	5.73	4.16	^*^	F108	0.77
									−11.02	^**^	F109	6.09
									6.10	^**^	G108	1.61
*ePGT-2^*AM*^*	BMC210-BMD002	Pv02 (2.8–9.1)	*ePGT-5^*AM*^*	IAC044-IAC010	Pv05 (0.0–3.7)	5.83	1.63^**^	3.89		ns		
**VEGETATIVE GROWTH TRAITS**												
*eNPB-2^*AM*^*	PVESTBR001-PVESTBR164	Pv02 (47.1–58.7)	*eNPB-10^*AM*^*	BMB221-BMB152	Pv10 (43.5–52.0)	14.51	0.08^**^	4.73	0.07	^*^	F108	2.69
*eNPB-7^*AM*^*	PVESTBR158-BMB202	Pv07 (16.4–18.6)	*eNPB-9^*AM*^*	PVESTBR082-BM154	Pv09 (45.3–50.1)	11.07	−0.11^**^	3.87	−0.17	^**^	F108	5.32
*eLI-2^*AM*^*	PVESTBR164-PVM115	Pv02 (58.7–59.5)	*eLI-3^*AM*^*	BMC201-IAC024	Pv03 (50.2–61.3)	5.97	0.31^*^	1.16	−1.51	^**^	F109	1.51
*eLI-3^*AM*^*	BMC201-IAC024	Pv03 (50.2–61.3)	*eLI-9^*AM*^*	BMB264-PVESTBR073	Pv09 (61.8–73.9)	6.93	−1.03^**^	2.13	1.34	^**^	G108	0.76
									0.85	^*^	F108	0.60
									−0.96	^*^	G108	0.52
**PLANT PRODUCTION TRAITS**												
*eBWI-5.1^*AM*^*	IAC044-IAC010	Pv05 (0.0–3.7)	*eBWI-9.1^*AM*^*	BMC221-BMB493	Pv09 (92.9–108.3)	4.97	−0.09^**^	6.34		ns		
*eBWI-5.2^*AM*^*	PVESTBR078-PVESTBR241	Pv05 (29.6–33.5)	*eBWI-9.2^*AM*^*	BMB563-PVESTBR166	Pv09 (122.7–125.1)	8.52	−0.13^**^	10.82		ns		
*eLL-7^*AM*^*	BMC294-BMC248	Pv07 (63.0–64.5)	*eLL-9^*AM*^*	BMC318-IAC019	Pv09 (129.9–134.3)	5.04	−0.02^**^	4.64	−0.03	^**^	G109	4.94
*eLWI-2^*AM*^*	PVESTBR008-BM172	Pv02 (112.7–122.8)	*eLWI-9^*AM*^*	BMC184-BM141	Pv09 (33.6–44.6)	5.73	−0.02^**^	6.59		ns		
*ePL-3^*AM*^*	BM282-BMD036	Pv03 (88.1–99.4)	*ePL-5.1^*AM*^*	PVESTBR078-PVESTBR241	Pv05 (29.6–33.5)	5.76	−0.77^**^	2.89	0.85	^**^	F108	2.90
*ePL-5.2^*AM*^*	PVESTBR241-BM142	Pv05 (33.5–42.7)	*ePL-10^*AM*^*	BMB221-BMB152	Pv10 (43.5–52.0)	5.46	0.40^**^	3.65	−0.37	^*^	F108	1.99
*eSL-4^*AM*^*	X04001-PVESTBR029	Pv04 (14.3–29.1)	*eSL-10^*AM*^*	BMB447-BM277	Pv10 (36.1–40.1)	7.32	0.66^**^	8.78		ns		
*eST-1^*AM*^*	BMD045-*FIN*	Pv01 (73.2–76.9)	*eST-4^*AM*^*	BMD016-BMC168	Pv04 (94.0–104.4)	7.83	0.13^**^	5.09	−0.13	^*^	G108	1.84
*eST-7^*AM*^*	IAC016-BMB621	Pv07 (27.8–31.9)	*eST-11^*AM*^*	BM240-PVESTBR057	Pv11 (10.2–18.3)	6.97	0.19^**^	12.20		ns		
*eSW-1^*AM*^*	IAC089-PvM120	Pv01 (40.0–45.7)	*eSW-10^*AM*^*	BMB447-BM277	Pv10 (36.1-40.1	6.71	−0.23^**^	10.75		ns		
*eSW-2^*AM*^*	BMB097-BMB080	Pv02 (28.4–32.0)	*eSW-3^*AM*^*	BMB506-BMC259	Pv03 (36.6–41.8)	5.86	0.19^**^	4.65		ns		
*eNSP-2^*AM*^*	BM172-BM237	Pv02 (122.8–125.0)	*eNSP-3^*AM*^*	BM098- BM282	Pv03 (87.7–88.1)	6.64	0.66^**^	10.10	−0.38	^*^	G108	1.88
*eNSP-7^*AM*^*	BMB621-BM210	Pv07 (31.9–38.0)	*eNSP-11^*AM*^*	PVM98-BMB428	Pv11 (68.9–78.5)	6.94	0.19^**^	4.53		ns		
*eNPP-2^*AM*^*	BM156-PVESTBR006	Pv02 (10.2–15.7)	*eNPP-5.2^*AM*^*	PVESTBR078-PVESTBR241	Pv05 (29.6–33.5)	9.01	−0.18^*^	1.51	−0.31	^*^	F109	1.18
*eNPP-5.1^*AM*^*	IAC044-IAC010	Pv05 (0.0–3.7)	*eNPP-8^*AM*^*	PVESTBR010-BMB445	Pv08 (18.8–30.2)	7.23	−0.39^**^	2.97	0.53	^**^	F108	1.82
									−1.29^**^	^**^	F109	12.45
*eNPP-6^*AM*^*	BMB419-BMD011	Pv06 (20.4–27.5)	*eNPP-10.1^*AM*^*	BMB447-BM277	Pv10 (36.1–40.1)	11.94	−0.38^**^	2.27	−0.68	^**^	F109	3.41
*eNPP-7^*AM*^*	BMB202-PVESTBR025	Pv07 (18.7–23.2)	*eNPP-10.2^*AM*^*	BMc150-X63525	Pv10 (47.4–53.8)	10.09	0.53^**^	2.51	1.47	^**^	F109	8.28
									−0.59	^*^	G108	1.27
*eSY-1^*AM*^*	*FIN*-PVESTBR268	Pv01 (76.9–78.6)	*eSY-10^*AM*^*	BMB447-BM277	Pv10 (36.1–40.1)	5.7	2.50^**^	1.70		ns		
*eSY -7^*AM*^*	BMB202-PVESTBR025	Pv07 (18.7–23.2)	*eSY-10^*AM*^*	BMB447-BM277	Pv10 (36.1–40.1)	7.55	3.63^**^	4.69	−3.31	^**^	F108	1.10
									−3.55	^**^	G108	0.18

a *E-QTLi and E-QTLj are the two QTLs involved in epistatic interaction*.

b *Linkage group and the estimated confidence interval of QTL position in the consensus map (in Kosambi cM)*.

c *F-values of significance of each QTL*.

d *Estimated additive by additive epistatic effect. Positive values indicate that alleles from BELUGA have a positive effect on the traits, and negative values indicate that positive effect on the traits is due to the presence of the alleles from PHA0399*.

e *Percentage of the phenotypic variation explained by additive by additive epistatic effects*.

f *Predicted additive by additive epistatic effect by environment interaction effect. The meaning of sign values is described in the third footnote (^d^)*.

g *Percentage of the phenotypic variation explained by additive by additive epistatic effect by environment interaction effect*.

* *P ≤ 0.05*,

** *P ≤ 0.01. Experiment wide P-value. Only significant effects are listed. ns, not significant effects on the four environments evaluated*.

*FT, flowering time; PGT, pod green time; NPB, the number of primary stem branches; LI, internode length; BWI, bracteole width; LL, leaflet length; LWI, leaflet width; PL, pod length; SL, seed length; ST, seed thickness; SW, 100 seed weight; NSP, number of seeds per pod; NPP, number of pods per plant; SY, seed yield*.

For flowering and maturity time traits, four epistatic interactions were identified in the MA RIL population (Table 4) that explained a phenotypic variance that ranged from 1.09 (*ePGT-2*^*MA*^ × *ePGT -10*^*MA*^) to 3.82% (*eFT-2.1*^*MA*^ × *eFT-10*^*MA*^). For the AM RIL population (Table 5), two epistatic interactions were detected whose effects accounting for up to 5.73% (*eFT-7*^*AM*^ × *eFT-9*^*AM*^) of the phenotypic variance. Among the six epistatic interactions detected for both RIL populations, three showed significant environmental interactions (E-QE) effects that ranged from 0.72 (*ePGT-2*^*MA*^ × *ePGT-10*^*MA*^ in G109) to 6.09% (*eFT-7*^*AM*^ × *eFT-9*^*AM*^ in F109).

Regarding vegetative growth traits, only one epistatic interaction was identified in the MA RIL population (Table 4), which did not show E-QE effects and explained the 2.34% of the phenotypic variance for the LI trait; whereas four epistatic interactions were detected in the AM RIL population (Table 5) whose effect ranged from 1.16 (*eLI-2*^*AM*^ × *eLI-3*^*AM*^) to 4.73% (*eNPB-2*^*AM*^ × *eNPB-10*^*AM*^). These four epistatic interactions displayed E-QE effects that explained up to 5.32% (*eNPB-7*^*AM*^ × *eNPB-9*^*AM*^ in F108) of the phenotypic variance.

For plant production traits, 12 epistatic interactions were identified in the MA RIL population (Table 4) and their additive by additive epistatic effects ranged from 1.11 (*ePWI-2*^*MA*^ × *ePWI-7*^*MA*^) to 8.30% (*eSL-1.1*^*MA*^ × *eSL-2*^*MA*^). Four out of the 12 epistatic interactions showed significant environment interaction effects that explained up to 1.29% (*eSW-6*^*MA*^ × *eSW-11*^*MA*^ in F109) of the phenotypic variance. For the AM RIL population (Table 5), 19 epistatic interactions were detected whose effects ranged from 1.51 (*eST-7*^*AM*^ × *eST-11*^*AM*^) to 12.20% (*eNPP-2*^*AM*^ × *eNPP-5.2*^*AM*^). Ten out of the 19 epistatic interactions displayed significant environment interactions and their effects ranged from 1.18 (*eSY-7*^*AM*^ × *eSY-10*^*AM*^ in G108) to 12.45% (*eNPP-5.1*^*AM*^ × *eNPP-8*^*AM*^ in F109).

## Discussion

Many traits of agronomic or biological importance undergo dynamic phenotypic changes during vegetative growth. In fact, the temporal control of flowering initiation determines the time invested in vegetative growth and, consequently, the vegetative resources available during reproduction. In addition, the rate of leaf, flower, and fruit production is a major component of vegetative growth. There is a close association between flowering initiation and vegetative growth; however, how these processes are coordinated during plant development remains unexplored. In this study, the variation for vegetative growth, the rate of leaf, flower, and fruit production, alongside their relationships with flowering and fruit time were investigated in two RIL populations from inter-gene pool crosses of an indeterminate Mesoamerican race Durango and a determinate Andean cultivar race Nueva Granada with the aim to unravel the genetic dynamics underlying vegetative growth and time to flowering.

### The genetic architecture of vegetative growth and flowering time in common bean

A prerequisite for QTL mapping is the assessment of the quantitative trait in multiple environments. In this study, agronomic evaluation was assessed in four different environments, under open field and greenhouse conditions. The analysis of the two RIL populations under these environments indicates predominantly quantitative inheritance rather than qualitative genes controlling vegetative growth and time to flowering. However, not only additive main effects are responsible for the phenotypic variation observed in our RIL mapping populations, but also epistatic interactions play an important role on the genetic control of flowering time and rate of plant production traits.

In both MA and AM RIL populations, normal distributions were found for most traits, although it was detected that the length of main stem could be controlled by a single gene in the AM RIL population since a bimodal distribution was found in the determinate type I Andean × indeterminate type IV Mesoamerican cross. This bimodal trend is adjusted with the discrete classes and the proportion expected for an autosomal major gene. Simple and complex genetic model has previously been proposed for plant height in common bean. Thus, Kornegay et al. (1992) observed that plant height is a trait of simple inheritance and high heritability. However, Frazier et al. (1958) stated that to reach again the erect trait in a plant of typically determinate growth habit, in addition to the *FIN* locus, it is needed the action of at least three recessive genes, or a set of minor action genes. Likewise, Davis and Frazier (1966) predicted several genes for internode length, as well as Checa et al. (2006) in indeterminate/indeterminate crosses of Andean and Mesoamerican beans; these authors found that the inheritance of plant height and internode length was mostly additive with only a few genes involved in the expression, and that these genes were most likely modified by interaction with minor genes and with the environment. Checa and Blair (2008) observed a quantitative inheritance rather than qualitative genes controlling plant height in an indeterminate type IV Mesoamerican × indeterminate type II Andean cross, although they also detected a relatively major gene or single locus with pleiotropic effects on plant height and internode length.

Transgressive segregation in both directions was observed for most of the evaluated traits in both RIL populations, except for the tendency of most lines to present averages for LMS and LI traits in the MA RIL population, and for SY and NPP traits in both RIL populations closer to the determinate Andean genotype, which might be related to segregation distortion. Thus, positive and negative transgressive segregations observed suggest that parental lines bear alleles that contribute to vegetative growth and time to flowering variation at several loci. High and significant heritability estimates, as well as positive correlations between LMS with FT and rate of plant production were found. FT was associated with initiation to immature or green pod and physiological pod or dry pod. In spite of the fact that FT and PST correlated with rate of plant production traits, it was not found correlation with PGT. Tar'an et al. (2002) also revealed positive correlations between components of plant height with FT and rate of seed production. Likewise, Koinange et al. (1996) reported that the gene *FIN* controlling determinacy has pleiotropic effects on FT, PST, and rate of plant production. In this work, it is shown that there is a cause-effect relation among LMS, FT, and rate of plant production traits, suggesting that physically linked or pleiotropic genes might be involved in the regulation of these traits (Aastveit and Aastveit, 1993).

Most of the single-locus QTLs detected in this work overlap with QTLs identified in some quantitative analyses of flowering time and vegetative growth carried out in common bean (Blair et al., 2006, 2010; Checa and Blair, 2008; Wright and Kelly, 2011). In general, these single-locus QTLs were responsible for the majority of the genetic variation for rate of plant production and time to flowering traits within common bean populations. However, it should be noted the presence of a considerable number of epistatic interactions for vegetative growth, plant production and flowering time traits that have not been reported so far. Taken together, these epistatic interactions reinforce the hypothesis that epistasis is involved in the genetic control of agronomical traits as well as epistatic interactions are more frequent in inter-gene pool crosses than in intra-gene pool crosses of common bean (Borel et al., 2016). Thus, for example, significant genetic interactions were found for the genomic region where the *FIN* locus is located (Pv01) with other genomic regions on Pv02, Pv06, and Pv11 in the MA RIL population, as well as on Pv04 in the AM RIL population. Epistatic interactions for flowering time traits were also detected in other genomic regions, whose effects explained up to 5.73% (*eFT-7*^*AM*^ and *eFT-9*^*AM*^ on Pv07 and Pv09, respectively) of the phenotypic variance.

Seed yield is mainly determined by factors such as number of seeds per pod and seed weight. In this study, the skewness to the lower values shown by the two related-productivity traits, SY and NPP, is in agreement with previous studies (Singh and Urrea, 1995; Johnson and Gepts, 1999; Bruzi et al., 2007), where the biological constraints of the inter-gene pool crosses, Andean and Mesoamerican, hamper reaching the maximum possible yields. Said limitation might result from the loss of favorable epistatic combinations or low probability of recovering superior genotype combination, among other reasons (Johnson and Gepts, 2002; Moreto et al., 2012). According to this hypothesis, the high number of epistatic interactions detected for all yield components is remarkable. Thus, epistatic interaction effects accounted for more than 10% of the phenotypic variance for PL (12.54%) and SL (13.7%) traits in the MA RIL population, as well as for ST (17.29%), SW (15.4%), and NSP (14.63%) traits in the AM RIL population. Hence, results of this research showed the importance of the epistatic effects in the genetic regulation of yield component traits. Thereby, both main and epistatic interaction effects should be considered for a successful application of marker assisted selection (MAS) programs in order to increase yield in common bean.

### Rate of plant production and time to flowering genetic links

In order to determine the genetic basis of the rate of plant production during the vegetative growth and time to flowering, QTL mapping was carried out with the average traits estimated in different conditions from flower to seed components. In both MA and AM RIL populations, it was detected a QTL located on Pv01 at the *FIN* locus, which showed large additive relative effects on time to flowering traits (up to 19% for FT and 32% for PST of the phenotypic variation in the MA and AM RIL populations, respectively). Comparative QTL analyses showed that this genomic region on Pv01 was also involved in the regulation of vegetative growth traits. Thus, QTLs for LMS, NPB, and LI traits were detected, which explained large additive effects (50–66% of the phenotypic variance for LMS). Within this genomic region, additional QTLs controlling pod size and productivity components (NSP and NPP) which explained more than 8% of the phenotypic variance. Furthermore, in both RIL populations, the *FIN* locus also displayed large effects on seed yield (19 and 15% of the phenotypic variance for SY trait in the MA and AM RIL populations, respectively). In the MA RIL population, the *FIN* locus also affected bracteole, leaf, pod, and seed size with a small to moderate additive effect (5 QTLs up to 3% of the phenotypic variance); whereas in AM RIL population, it affected pod size (9% of the phenotypic variance for PWI). In addition to the *FIN* locus, other genomic regions were also involved in the regulation of rate of plant production and time to flowering traits, although with a minor effect. Thus, for example, QTLs for PGT and PST in the MA population and for PT in the AM population were detected on Pv02 (PVESTBR006-GATS91), which colocalized with a QTL for seed weight previously mapped by Blair et al. (2006). Taken together, comparative QTL analysis results indicated that vegetative growth has a large effect on time to flowering and the rate of plant production traits, explaining the pleiotropic effects observed for FT and LMS traits. It is mainly due to the phenotypic effect of the recessive allele at the *FIN* locus, which is present in the determinate type I genotype (Beluga) and controls the meristem switch from a vegetative to a reproductive state. The *fin* allele reduces the plant growing period, causing a reduction in length of the main stem and number of branches, and an increase in internode length, as well as small bracteoles and leaves, large pods, and seeds that give rise to a lower yield (Norton, 1915; Ojehomon and Morgan, 1969; Koinange et al., 1996), resulting in common bean cultivars with a reduced flowering period since they mature more rapidly (Cober and Tanner, 1995; Koinange et al., 1996).

### Genetic and molecular mechanisms underlying the link between time to flowering and vegetative growth

Currently, there are genes underlying flowering time QTLs which have been isolated in other crops; said crops have a growth pattern similar to common bean through successive series of modular units. In a common bean plant with a determinate type I growth habit, after floral initiation, the terminal shoot meristem produces a terminal inflorescence and ceases its vegetative growth. However, in a common bean plant exhibiting an indeterminate type IV growth habit, the terminal shoot meristem produces stem nodes, each one composed by one compound leaf and an inflorescence in its axil; thus, vegetative, and reproductive structures are continuously produced until maturity and senescence. This growth pattern through successive series of modular units is similar to that of tomato (Sage and Webster, 1987; Schmitz and Theres, 1999). In tomato, *SELF-PRUNING* (*SP*) and *FALSIFLORA* (*FA*) control meristem identity. *SP* gene suppresses the transition of vegetative to reproductive state, keeping a plant indeterminate (Pnueli et al., 1998). *FA* is responsible for floral meristem identity and promotes flowering (Molinero-Rosales et al., 1999). *SP* is the homolog of the *Antirrhinum majus CENTRORADIALIS (CEN)* and *A. thaliana TERMINAL FLOWER1* (*TFL1*) genes (Pnueli et al., 2001). *FA* is an ortholog of *A. thaliana LFY* and *A. majus FLORICAULA* (*FLO*). *LFY* in *A. thaliana* activates directly *AP1*, causing flowering (Komeda, 2004; Saddic et al., 2006). Foucher et al. (2003) found that a pea homolog of the *A. thaliana TFL1, PsTFL1a*, corresponds to the determinacy locus (*DETERMINATE*; *DET*), and that another pea *TFL1* homolog, *PsTFL1c* (*LATE FLOWERING*; *LF*), acts as a repressor of flowering. Likewise, Mir et al. (2014) revealed the orthologous nature of *CcTFL1* gene for determinacy in pigeonpea; whereas in soybean, Tian et al. (2010) showed evidence that *Dt1* (*GmTFL1*) is a homolog of the Arabidopsis *TFL1* gene, which has a high-level of conservation with the common bean *PvTFL1y* gene. This gene has been proposed as a candidate gene for the *FIN* locus since mutations at the *PvTFL1y* locus were found to cosegregate with the determinate growth habit phenotype (Repinski et al., 2012). In this study, the position of *PvTFL1y* (*Phvul.001G189200*) was found within the QTLs positioned in the marker intervals of *FIN*-BMC224 and BMD045-*FIN* on Pv01. This finding is consistent with evidence at Andean *PvTFL1y* haplotype of the determinacy locus (Repinski et al., 2012). However, more information is needed to know whether different *PvTFL1y* haplotypes derived independently in each gene pool or whether the determinacy locus arose in a single gene pool, as happens in rice. In this species, the determinacy locus arose in a unique gene pool (*indica* or *japonica*) and was later transferred to the other pools (Sweeney et al., 2007). Furthermore, the correlated effects of the *PvTFL1y* locus on other plant traits, such as length of main stem or internode length, and productivity have been demonstrated in this work.

## Future remarks

The information herein reported could be used not only to establish different breeding strategies combining loci from the different gene pools of common bean, but also to look for associations of genetic variation in determinacy candidate genes in other legume crops with varieties bred for determinate growth habit, such as *P. coccineus* (runner bean), *P. lunatus* (lima bean), and *Cajanus cajan* (pigeonpea) (Waldia and Singh, 1987; van Rheenen et al., 1994; Huyghe, 1998). Furthermore, exploring the interaction and linkage of loci for vegetative growth, flowering time, and the rate of plant production may allow for the expansion of common bean to the geographic locations in which novel adaptation traits can be evaluated.

## Author contributions

AG carried out molecular marker and QTL analysis and drafted the manuscript. FY supported mapping methodologies and contributed to a critical review of the manuscript. SS performed the phenotypic data collection. SB contributed to molecular marker analysis. AD contributed to the review of the manuscript. RL collaborated in experimental design and critical review of the manuscript. MS planned the research work, assisted in analysis and interpretation of the data, and edited the manuscript. All authors have read and approved the final version of the manuscript.

### Conflict of interest statement

The authors declare that the research was conducted in the absence of any commercial or financial relationships that could be construed as a potential conflict of interest.
