# Supplementary material for: Major Contribution of Flowering Time and Vegetative Growth to Plant Production in Common Bean As Deduced from a Comparative Genetic Mapping
Source: Front Plant Sci. 2016 Dec 26;7:1940. doi: 10.3389/fpls.2016.01940 (PMC5183638; doi:10.3389/fpls.2016.01940)
Supplement: Supplementary file 1 [file Table1.PDF]

Supplementary Table 1. Summary and description of traits measured

Trait	Ab.[a]	Unit	Description
**Flowering and maturity traits**			
Days to flowering	FT	days	recorded when a plant had one open flower on a primary branch
Days to young-green pod	PGT	days	recorded from planting to 50% of the immature pods on a plot basis
Days to physiological pod maturity	PST	days	recorded when a plant had one dry pod on a primary branch
**Vegetative growth traits**			
Length of main stem	LMS	cm	distance measured from the base at ground level to the uppermost leaflet of the longest branch; average of 10 plants
Number of primary stem branches	NPB	count	stems winding around the support strings halfway up the length of the plant; average of 10 plants
Internode length	LI	cm	length of the fifth internode on the main stem; average of 10 plants
**Plant production traits**			
Bracteole length	BL	mm	distance measured along the midrib of the lamina; average of 10 flowers
Bracteole width	BWI	mm	distance measured between the widest lobes of the lamina perpendicular to the lamina mid-rib; average of 10 flowers
Leaflet length	LL	cm	distance measured from the lamina tip to the point of petiole intersection along the midrib; average of 10 leaflets
Leaflet width	LWI	cm	distance measured at the widest point perpendicular to the midrib; average of 10 leaflets
Pod length	PL	mm	distance measured from the peduncle connection point to the apex excluding the beak; average of 10 pods
Pod width	PWI	mm	distance measured at right angles to the sutures at the level of the second seed from the apex; average of 10 pods
Pod thickness	PT	mm	distance measured between sides at the level of the second and third seed from the apex; average of 10 pods
Seed length	SL	mm	the longest distance across the seed parallel to the hilum; average of 10 seeds
Seed width	SWI	mm	the longest distance across the seed perpendicular to the hilum; average of 10 seeds
Seed thickness	ST	mm	the longest distance from top to bottom of the seed; average of 10 seeds
See weight	SW	g 100 seeds-1	determined on 100 dry seeds per plot
Number of seeds per pod	NSP	count	average number of 10 pods
Number of pods per plant	NPP	count	average number of 10 plants
Seed yield	SY	kg ha-1	weight of dried seed (at 14% moisture content)

[a]Ab. Trait abbreviations
