# Supplementary material for: Major Contribution of Flowering Time and Vegetative Growth to Plant Production in Common Bean As Deduced from a Comparative Genetic Mapping
Source: Front Plant Sci. 2016 Dec 26;7:1940. doi: 10.3389/fpls.2016.01940 (PMC5183638; doi:10.3389/fpls.2016.01940)
Supplement: Supplementary file 2 [file Table2.PDF]

Supplementary Table 2. Mean values, standard errors, range of variation and variance analysis of the quantitative traits analysed in the two common bean parents, PHA0419 and BELUGA, and the MA RIL population grown in four different environments (Env).

Trait[a]	Env[b]	Block	Parents			RILs			
			PHA0419 ± SE	BELUGA ± SE	P_{PAR}	N[c]	Mean ± SE	Range	P_{RIL}
**Flowering and maturity traits**									
FT (days)	F108	ns	49.5 ± 0.49	32.5 ± 0.20	**	178	39.8 ± 0.26	30.0 - 52.0	**
	F109	ns	51.5 ± 0.86	33.0 ± 0.98	**	158	41.2 ± 0.36	30.5 - 70.0	**
	G108	ns	47.8 ± 0.89	30.7 ± 0.93	**	179	42.6 ± 0.34	31.6 - 58.5	**
	G109	ns	55.0 ± 0.87	29.7 ± 0.23	**	177	37.9 ± 0.43	20.0 - 58.5	**
PGT (days)	F108	ns	68.5 ± 0.19	57.5 ± 0.26	*	175	64.1 ± 0.21	56.0 -702.5	**
	F109	ns	73.0 ± 0.59	65.0 ± 0.29	*	120	66.4 ± 0.38	58.0 - 84.0	**
	G108	*	64.7 ± 0.39	55.0 ± 0.36	**	179	61.7 ± 0.41	47.7 - 79.3	**
	G109	ns	65.0 ± 0.58	43.5 ± 0.46	**	177	58.3 ± 0.56	38.5 - 84.0	**
PST (days)	F108	ns	86.5 ± 0.46	81.0 ± 0.23	ns	174	78.7 ± 0.38	61.0 - 95.5	**
	F109	ns	89.0 ± 0.59	79.0 ± 0.46	*	86	84.7 ± 0.67	59.0 - 99.5	**
	G108	ns	94.3 ± 0.26	85.0 ± 0.32	**	179	90.5 ± 0.43	78.2 - 106.0	**
	G109	ns	94.0 ± 0.38	88.0 ± 0.51	*	177	90.0 ± 0.64	68.0 - 117.0	**
**Vegetatibe growth traits**									
LMS (cm)	F108		NE	NE			NE		
	F109		NE	NE			NE		
	G108	ns	281.5 ± 4.63	48.6 ± 1.99	**	179	136.1 ± 6.04	19.1 - 341.7	**
	G109	ns	285.6 ± 3.26	55.3 ± 2.36	**	175	145.0 ± 5.97	23.0 - 337.5	**
NPB	F108		NE	NE			NE		
	F109		NE	NE			NE		
	G108	ns	1.5 ± 0.01	3.2 ± 0.02	*	179	1.9 ± 0.04	1.0 - 3.2	**
	G109	ns	1.2 ± 0.01	3.0 ± 0.03	*	175	1.8 ± 0.04	1.0 - 3.5	**
	F108	ns	13.1 ± 0.12	6.2 ± 0.21	**	179	4.4 ± 0.10	2.0 - 12.0	**
LI (cm)	F109	ns	15.5 ± 0.17	4.9 ± 0.02	**	158	3.7 ± 0.08	1.5 - 17.5	**
	G108	*	15.8 ± 0.26	6.5 ± 0.19	**	179	11.1 ± 0.31	3.8 - 24.1	*
	G109	ns	16.2 ± 0.28	4.9 ± 0.16	**	177	9.2 ± 0.21	1.9 - 17.7	*

**Plant production traits**									
BL (mm)	F108	ns	5.6 ± 0.04	4.2 ± 0.03	*	178	4.9 ± 0.06	3.0 - 7.8	**
	F109	ns	6.2 ± 0.08	5.1 ± 0.05	*	158	5.1 ± 0.06	3.1 - 7.7	*
	G108	ns	6.5 ± 0.07	5.1 ± 0.06	**	179	5.3 ± 0.07	3.7 - 9.0	**
	G109	ns	6.2 ± 0.06	5.0 ± 0.05	**	178	6.2 ± 0.07	4.0 - 9.2	**
BWI (mm)	F108	ns	4.3 ± 0.03	3.1 ± 0.02	**	178	3.3 ± 0.05	2.0 - 5.6	*
	F109	ns	5.2 ± 0.03	3.9 ± 0.06	**	158	3.4 ± 0.05	1.9 - 5.0	**
	G108	ns	4.8 ± 0.06	3.5 ± 0.03	*	179	3.9 ± 0.06	2.4 - 6.6	**
	G109	ns	5.1 ± 0.04	4.2 ± 0.04	*	178	4.3 ± 0.06	2.0 – 6.5	**
LL (cm)	F108	ns	12.7 ± 0.06	8.5 ± 0.06	ns	179	8.7 ± 0.06	6.5 - 12.5	ns
	F109	*	14.0 ± 0.09	9.2 ± 0.09	ns	158	9.1 ± 0.06	7.0 - 11.0	ns
	G108	ns	13.0 ± 0.03	10.1 ± 0.20	*	179	11.1 ± 0.15	6.6 - 19.5	*
	G109	ns	15.1 ± 0.12	10.2 ± 0.08	*	179	9.8 ± 0.09	7.0 - 13.1	*
LWI (cm)	F108	ns	7.5 ± 0.16	5.5 ± 0.09	*	179	6.6 ± 0.06	5.0 - 9.5	*
	F109	*	7.3 ± 0.10	5.7 ± 0.08	ns	158	6.5 ± 0.06	5.0 - 9.5	*
	G108	ns	7.6 ± 0.13	5.6 ± 0.12	*	179	7.9 ± 0.12	4.6 - 14.3	*
	G109	ns	7.5 ± 0.12	5.9 ± 0.11	ns	179	7.2 ± 0.08	4.6 - 11.0	*
PL (mm)	F108	*	154 ± 1.0	121 ± 5.0	**	174	110 ± 1.0	65 - 144	**
	F109	ns	135 ± 2.0	110 ± 1.2	*	86	100 ± 1.8	55 - 135	**
	G108	ns	140 ± 1.0	117 ± 0.9	**	179	115 ± 1.1	79 - 166	**
	G109	ns	127 ± 1.1	114 ± 1.0	ns	177	114 ± 1.1	76 - 189	**
PWI (mm)	F108	ns	11.8 ± 0.04	13.6 ± 0.06	ns	174	11.4 ± 0.07	9.0 - 14.9	**
	F109	ns	12.4 ± 0.12	13.5 ± 0.14	**	86	10.6 ± 0.15	7.6 - 15.5	**
	G108	*	11.2 ± 0.09	14.0 ± 0.08	**	179	12.7 ± 0.11	8.8- 17.4	**
	G109	ns	12.5 ± 0.11	15.5 ± 0.10	ns	177	13.7 ± 0.12	8.0- 18.3	**
PT (mm)	F108	*	4.5 ± 0.04	6.1 ± 0.06	*	174	5.3 ± 0.05	3.5 - 7.9	*
	F109	ns	5.3 ± 0.09	7.8 ± 0.08	*	86	4.8 ± 0.15	2.9 - 9.4	**
	G108	ns	5.6 ± 0.05	7.4 ± 0.07	**	179	6.1 ± 0.07	3.8 - 8.2	*
	G109	ns	5.3 ± 0.06	7.6 ± 0.05	*	177	6.5 ± 0.07	4.7 - 10.4	**
SL (mm)	F108	ns	17.3 ± 0.09	15.1 ± 0.12	*	169	15.7 ± 0.11	11.2 - 19.0	**
	F109	ns	17.6 ± 0.12	14.1 ± 0.10	*	74	15.0 ± 0.18	11.4 - 19.3	**
	G108	ns	19.9 ± 0.10	16.9 ± 0.08	**	179	17.4 ± 0.12	13.1 - 21.3	**

	G109	ns	18.8 ± 0.08	16.0 ± 0.09	**	176	16.9 ± 0.13	12.8 - 20.7	**
SWI (mm)	F108	ns	10.2 ± 0.08	7.7 ± 0.03	*	169	8.4 ± 0.05	6.0 - 10.6	**
	F109	ns	10.9 ± 0.11	8.0 ± 0.08	**	74	7.9 ± 0.13	5.9 - 9.6	**
	G108	ns	11.2 ± 0.07	8.6 ± 0.05	**	179	9.5 ± 0.06	7.8 - 11.8	**
	G109	ns	10.8 ± 0.09	8.7 ± 0.04	*	176	9.4 ± 0.06	7.5 - 11.7	**
ST (mm)	F108	ns	5.1 ± 0.02	6.5 ± 0.03	*	169	5.6 ± 0.04	3.9 - 6.8	*
	F109	ns	5.2 ± 0.05	6.8 ± 0.09	*	74	5.3 ± 0.11	3.8 - 7.0	**
	G108	ns	5.6 ± 0.03	6.9 ± 0.03	**	179	6.2 ± 0.05	4.8 - 7.9	*
	G109	ns	5.4 ± 0.04	7.1 ± 0.04	**	176	6.0 ± 0.05	3.9 - 7.7	**
SW (g 100 seeds^{-1})	F108	ns	81.0 ± 0.56	59.1 ± 0.52	**	169	46.2 ± 0.98	23.5 - 123.1	**
	F109	ns	82.5 ± 0.53	52.0 ± 0.48	**	73	40.8 ± 1.89	19.0 - 73.6	**
	G108	ns	91.5 ± 0.63	63.5 ± 0.45	**	179	68.7 ± 0.97	33.0 - 102.0	**
	G109	ns	79.5 ± 0.48	56.2 ± 0.66	*	176	60.9 ± 0.94	26.2 - 92.9	**
NSP	F108	*	3.2 ± 0.03	3.7 ± 0.25	**	169	2.9 ± 0.05	1.2 - 4.5	**
	F109	ns	5.1 ± 0.06	3.2 ± 0.25	*	74	2.0 ± 0.10	1.0 - 3.5	*
	G108	*	3.4 ± 0.05	3.5 ± 0.27	*	179	3.5 ± 0.05	1.2 - 5.3	**
	G109	ns	5.0 ± 0.04	4.0 ± 0.29	**	176	3.4 ± 0.06	1.0 - 5.2	**
NPP	F108	*	26.0 ± 0.56	10.0 ± 1.02	**	169	15.2 ± 0.96	1.0 - 75.0	**
	F109	ns	28.5 ± 1.62	12.3 ± 0.92	**	74	23.3 ± 0.50	1.0 - 82.0	**
	G108	ns	36.3 ± 0.89	14.3 ± 0.96	**	179	26.4 ± 1.23	3.3 - 83.7	**
	G109	ns	37.0 ± 1.93	11.5 ± 1.01	**	176	28.2 ± 1.50	2.0 - 122.0	**
SY (kg ha^{-1})	F108	*	2052 ± 42.5	778 ± 32.1	**	118	1591± 50.2	21 - 2596	**
	F109		NE	NE			NE		
	G108	*	3515 ± 36.9	1048 ± 54.6	**	179	1851 ± 96.8	129 - 7427	**
	G109	*	3131 ± 46.8	663 ± 45.6	**	176	1815 ± 116.1	102- 7763	**

ns, no significant differences, NE not evaluated.
*, ** Significant at the 0.05 and 0.01 probability levels, respectively, for differences among blocks, parents (P_{PAR}) and RILs (P_{RIL}).
[a] FT: days to flowering; PGT: days to immature pod harvest; PST: days to physiological maturity; LMS: length of main stem; NPB: the number of primary stem branches; LI: internode length; BL: bracteole length; BWI: bracteole width; LL: leaflet length; LWI: leaflet width; PL: pod length; PWI: pod width; PT: pod thickness; SL: seed length; SWI: seed width; ST: seed thickness; SW: 100 seed weight; NSP: number of seeds per pod; NPP: number of pods per plant; SY: seed yield.
[b] Sowing dates of experiments were April 2, 2008 (F108 code), May 8, 2009 (F109 code), March 25, 2008 (G108), February 9, 2009 (G109 code).
[c] N number of lines recorded.
