# Supplementary material for: Major Contribution of Flowering Time and Vegetative Growth to Plant Production in Common Bean As Deduced from a Comparative Genetic Mapping
Source: Front Plant Sci. 2016 Dec 26;7:1940. doi: 10.3389/fpls.2016.01940 (PMC5183638; doi:10.3389/fpls.2016.01940)
Supplement: Supplementary file 3 [file Table3.PDF]

Supplementary Table 3. Mean values, standard errors, range of variation and variance analysis of quantitative traits analysed in the two common bean parents, BELUGA and PHA0399, and the AM RIL population grown in four different environments (Env).

**Trait**[a]	**Env**[b]	**Block**	**Parents**			**RILs**			
			**BELUGA ± SE**	**PHA0399 ± SE**	**P_{PAR}**	**N**[c]	**Mean ± SE**	**Range**	**P_{RIL}**
**Flowering and maturity traits**									
FT (days)	F108	ns	24.1 ± 0.35	42.8 ± 0.65	**	58	39.7 ± 0.63	31.0 - 74.0	**
	F109	ns	29.0 ± 0.30	35.4 ± 1.15	**	54	43.7 ± 1.58	21.7 - 95.8	**
	G108	*	30.1 ± 0.85	39.0 ± 0.55	**	49	37.6 ± 1.12	24.0 - 55.0	**
	G109	ns	32.6 ± 1.10	46.0 ± 0.20	**	60	39.4 ± 1.08	22.3 - 53.0	**
PGT (days)	F108	ns	54.8 ± 0.35	69.0 ± 1.05	*	58	61.8 ± 0.60	49.0 -70.0	**
	F109	*	57.4 ± 1.10	69.9 ± 1.35	**	52	61.7 ± 1.43	35.0 - 79.0	**
	G108	ns	48.0 ± 1.50	60.0 ± 0.50	**	49	55.9 ± 1.18	43.0 - 76.5	**
	G109	ns	48.9 ± 0.95	63.7 ± 0.25	**	60	60.9 ± 1.35	43.5 - 83.3	**
PST (days)	F108	ns	77.9 ± 0.40	102.1 ± 2.10	**	58	81.1 ± 0.87	68.0 - 96.0	**
	F109	ns	75.6 ± 0.55	97.4 ± 1.10	**	40	92.0 ± 0.87	75.3 - 111.0	**
	G108	ns	75.5 ± 0.95	88.9 ± 0.65	**	49	86.4 ± 1.18	71.0 - 115.0	**
	G109	*	79.1 ± 0.90	120.9 ± 1.60	**	60	107.5 ± 2.57	78.0 - 137.0	**
**Vegetative growth traits**									
LMS (cm)	F108		NE	NE			NE		
	F109	ns	55.2 ± 2.84	223.5 ± 23.5	**	54	175.4 ± 12.09	41.0- 267.0	**
	G108	ns	53.3 ± 2.66	286.7 ± 1.66	**	59	217.31 ± 14.37	49.7- 382.5	**
	G109	ns	62.5 ± 5.00	315.0 ± 12.50	**	59	216.6 ± 14.38	41.7 - 350.0	**
NPB	F108		NE	NE			NE		
	F109	ns	3.3 ± 0.34	2.1 ± 0.08	**	54	3.2 ± 0.08	2.0 - 4.5	**
	G108	ns	3.2 ± 0.17	2.8 ± 0.19	*	59	3.0 ± 0.08	1.5 - 4.7	**
	G109	ns	3.5 ± 0.01	2.6 ± 0.10	**	59	3.3 ± 0.11	1.7 - 5.0	**
LI (cm)	F108	ns	6.3 ± 0.38	15.2 ± 1.25	**	58	14.2 ± 0.17	2.9 - 11.5	**
	F109	ns	10.1 ± 0.13	22.6 ± 0.81	**	54	14.8 ± 0.55	5.9 - 22.3	**
	G108	ns	9.2 ± 0.25	18.7 ± 0.25	**	48	12.3 ± 0.68	3.2 - 22.0	**
	G109	*	8.0 ± 0.10	20.3 ± 0.97	**	59	18.2 ± 0.86	7.5 - 27.8	**

Plant production traits

BL (mm)	F108	ns	6.0 ± 0.40	5.9 ± 0.01	ns	58	5.2 ± 0.10	4.1 - 8.4	**
	F109	ns	6.9 ± 0.20	5.5 ± 0.57	**	54	7.1 ± 0.18	4.6 - 10.4	**
	G108	ns	6.1 ± 0.04	6.3 ± 0.53	ns	49	6.1 ± 0.21	3.8 - 9.7	**
	G109	ns	7.4 ± 0.36	5.2 ± 0.03	**	59	5.8 ± 0.12	4.0 - 8.1	**
BWI (mm)	F108	ns	4.1 ± 0.12	5.5 ± 0.50	**	58	3.7 ± 0.10	2.5 - 6.9	*
	F109	ns	4.6 ± 0.15	4.9 ± 0.39	*	54	5.1 ± 0.16	3.4 - 7.6	**
	G108	ns	3.8 ± 0.41	4.7 ± 0.09	**	49	4.7 ± 0.19	2.9 - 7.7	**
	G109	ns	4.6 ± 0.11	5.3 ± 0.34	*	59	4.4 ± 0.13	2.7 - 7.3	**
LL (cm)	F108	ns	9.0 ± 0.06	12.9 ± 0.56	**	58	9.3 ± 0.06	8.0 - 12.7	*
	F109	ns	15.7 ± 0.91	10.1 ± 0.20	**	53	14.7 ± 0.20	11.7 - 19.8	**
	G108	ns	11.7 ± 0.75	13.5 ± 0.02	*	49	11.4 ± 0.23	8.8 - 15.3	**
	G109	ns	12.1 ± 0.49	14.8 ± 0.42	**	59	14.0 ± 0.19	11.3 - 16.6	*
LWI (cm)	F108	ns	6.0 ± 0.06	10.0 ± 0.56	**	58	7.7 ± 0.11	5.5 - 12.0	*
	F109	ns	6.2 ± 0.91	11.2 ± 0.20	**	53	7.1 ± 0.16	7.0 - 13.0	**
	G108	ns	7.7 ± 0.75	11.9 ± 0.02	*	49	7.9 ± 0.15	6.0 - 11.5	**
	G109	ns	6.6 ± 0.49	14.8 ± 0.42	**	59	7.4 ± 0.18	6.8 - 14.1	*
PL (mm)	F108	ns	123 ± 1.0	109 ± 0.5	*	58	124 ± 01.5	102 - 147	**
	F109	ns	140 ± 0.9	114 ± 2.8	**	54	115 ± 2.2	72 - 146	**
	G108	ns	126 ± 1.5	117 ± 3.2	*	49	139 ± 2.2	113 - 172	*
	G109	ns	134 ± 4.3	109 ± 6.7	**	59	113 ± 1.5	87 - 135	*
PWI (mm)	F108	ns	13.2 ± 0.31	13.0 ± 0.03	ns	58	12.3 ± 0.13	10.0 - 14.0	**
	F109	ns	13.8 ± 0.22	11.8 ± 0.71	**	54	11.6 ± 0.18	8.4- 14.5	**
	G108	ns	15.2 ± 0.05	13.2 ± 0.05	**	49	13.2 ± 0.21	9.5 - 16.6	**
	G109	ns	15.3 ± 0.23	12.3 ± 0.23	**	59	13.7 ± 0.18	11.1 - 18.3	**
PT (mm)	F108	ns	6.8 ± 0.25	5.4 ± 0.11	*	58	5.5 ± 0.07	4.2 - 6.9	*
	F109	ns	7.1 ± 0.06	5.6 ± 0.07	**	54	6.0 ± 0.10	3.6 - 7.5	**
	G108	ns	7.0 ± 0.11	4.9 ± 0.04	**	49	5.6 ± 0.12	4.4 - 9.4	**
	G109	ns	7.8 ± 0.42	5.4 ± 0.10	**	59	6.7 ± 0.11	4.9 - 8.7	*
SL (mm)	F108	ns	15.9 ± 0.34	19.2 ± 0.50	**	57	15.9 ± 0.21	12.4 - 18.9	**
	F109	ns	16.8 ± 0.27	18.9 ± 0.58	*	54	16.9 ± 0.22	13.7 - 20.4	**
	G108	ns	14.3 ± 0.40	19.4 ± 0.55	**	48	16.2 ± 0.19	12.8 - 55.0	**

	G109	ns	16.1 ± 0.02	20.2 ± 0.57	**	59	17.7 ± 0.20	15.0 - 20.6	**
SWI (mm)	F108	ns	8.0 ± 0.03	10.7 ± 0.23	*	57	8.5 ± 0.11	6.9 - 10.7	**
	F109	ns	8.5 ± 0.27	10.7 ± 0.52	*	54	8.8 ± 0.09	7.1 - 10.6	*
	G108	ns	8.4 ± 0.26	11.0 ± 0.12	**	48	9.0 ± 0.10	7.6 - 10.6	*
	G109	ns	8.3 ± 0.48	11.5 ± 0.44	**	59	9.8 ± 0.11	8.0 - 11.6	**
ST (mm)	F108	ns	6.9 ± 0.03	6.4 ± 0.03	*	57	5.9 ± 0.06	4.7 - 7.1	*
	F109	ns	6.9 ± 0.04	5.6 ± 0.02	**	54	6.1 ± 0.0.06	5.0 - 7.2	*
	G108	ns	6.2 ± 0.36	6.8 ± 0.24	*	48	5.9 ± 0.09	3.9 - 7.2	**
	G109	ns	7.4 ± 0.01	7.2 ± 0.25	ns	59	6.6 ± 0.06	5.6 - 7.9	*
SW (g 100 $seeds^{-1}$)	F108	ns	60.5 ± 1.85	83.6 ± 1.59	**	57	53.3 ± 1.56	33.5 - 90.0	**
	F109	ns	65.7 ± 0.66	75.5 ± 0.90	**	54	60.1 ± 1.42	38.5 - 91.5	**
	G108	ns	65.5 ± 1.57	85.0 ± 1.00	**	48	57.7 ± 1.61	33.0 - 86.0	**
	G109	ns	60.7 ± 1.68	103.0 ± 6.03	**	59	79.2 ± 1.70	46.5 - 109.0	**
NSP	F108	*	4.4 ± 0.13	3.7 ± 0.25	**	57	3.0 ± 0.12	1.5 - 5.0	**
	F109	*	4.7 ± 0.25	3.5 ± 0.27	**	54	3.9 ± 0.15	1.5 - 5.8	**
	G108	ns	4.5 ± 0.01	3.2 ± 0.25	**	49	4.4 ± 0.29	3.0 - 5.5	*
	G109	ns	4.5 ± 0.01	3.5 ± 0.03	*	59	3.4 ± 0.12	1.2 - 5.5	**
NPP	F108	ns	30.0 ± 0.05	159.2 ± 2.75	**	57	30.4 ± 1.09	2.0 - 140.5	**
	F109	ns	25.8 ± 0.51	71.2 ± 5.2	**	54	50.7 ± 4.58	1.3 - 178.0	**
	G108	*	17.3± 1.67	86.7 ± 5.75	**	49	58.5 ± 7.24	6.0 - 163.0	**
	G109	ns	23.9 ± 0.35	70.8 ± 4.43	**	59	40.3 ± 2.41	10.0 - 84.0	**
SY (kg ha^{-1})	F108	*	1123 ± 133.2	3816 ± 707.3	**	41	920 ± 140.7	92 - 3970	**
	F109	*	1988 ± 35.5	4847 ± 363.2	**	55	3705 ± 387.5	55 - 13065	**
	G108	*	1505 ± 81.5	4640 ± 17.0	**	48	4081 ± 615.3	65- 13994	**
	G109	ns	1078 ± 88.2	3983 ± 17.1	**	60	1182 ± 72.3	201 - 2945	**

ns, no significant differences, NE not evaluated.
*, ** Significant at the 0.05 and 0.01 probability levels, respectively, for differences among blocks, parents (P_{PAR}) and RILs (P_{RIL}).
[a] FT: days to flowering; PGT: days to immature pod harvest; PST: days to physiological maturity; LMS: length of main stem; NPB: the number of primary stem branches; LI: internode length; BL: bracteole length; BWI: bracteole width; LL: leaflet length; LWI: leaflet width; PL: pod length; PWI: pod width; PT: pod thickness; SL: seed length; SWI: seed width; ST: seed thickness; SW: 100 seed weight; NSP: number of seeds per pod; NPP: number of pods per plant; SY: seed yield.
[b] Sowing dates of experiments were April 2, 2008 (F108 code), May 8, 2009 (F109 code), March 25, 2008 (G108), February 9, 2009 (G109 code).
[c] N number of lines recorded.
