# Supplementary material for: Major Contribution of Flowering Time and Vegetative Growth to Plant Production in Common Bean As Deduced from a Comparative Genetic Mapping
Source: Front Plant Sci. 2016 Dec 26;7:1940. doi: 10.3389/fpls.2016.01940 (PMC5183638; doi:10.3389/fpls.2016.01940)
Supplement: Supplementary file 4 [file Table4.PDF]

Supplementary Table 4. Phenotypic correlation coefficients and heritability estimates with their standard errors among agronomical traits in the MA (below the main diagonal) and AM (above the main diagonal) RIL populations.

	FT	PGT	PST	LMS	NPB	LI	BL	BWI	LL	LWI	PL	PWI	PT	SL	SWI	ST	SW	NSP	NPP	SY	h^2 ± S.E. (MA)	h^2 ± S.E. (AM)
**FT**		0.76 **	0.55 **	0.50 **	-0.43 **	0.33 **			0.21 *	0.17 *			0.16 *	0.16 *					0.19 *	0.16 *	0.65 ± 0.04	0.62 ± 0.03
**PGT**	0.57 **		0.63 **	0.42 **	-0.38 **	0.23 *		-0.17 *													0.50 ± 0.09	0.53 ± 0.02
**PST**	0.46 **	0.27 **		0.55 **	-0.43 **	0.68 **			0.50 **	0.51 **		0.38 **	0.35 **	0.38 **	0.52 **	0.35 **	0.49 **		0.34 **		0.47 ± 0.08	0.72 ± 0.01
**LMS**	0.38 *	0.21 **	0.23 **		-0.57 **	0.45 **		-0.26 *			0.37 **	0.36 **	0.30 **		0.31 **		0.23 *	0.41 **	0.46 **		0.86 ± 0.02	0.83 ± 0.07
**NPB**		-0.29 **		-0.16 *		-0.15 *		0.21 *			-0.24 *				-0.23 *			-0.24 *			0.47 ± 0.16	0.48 ± 0.05
**LI**		0.21 **	0.42 **	0.78 **	-0.59 **				0.60 **	0.61 **		0.24 **	0.45 **	0.40 **	0.47 **	0.39 **	0.53 **	0.31 **	0.46 **		0.49 ± 0.07	0.42 ± 0.03
**BL**		-0.14 *	0.25 **	0.26 **		0.26 **		0.87 **	0.30 **	0.31 **											0.40 ± 0.08	0.70 ± 0.05
**BWI**		-0.15 *	0.29 **	0.24 **		0.35 **	0.84 **		0.35 **	0.38 **											0.43 ± 0.07	0.47 ± 0.03
**LL**	0.13 *	-0.11 *	0.37 **	0.28 **		0.59 **	0.20 *	0.18 *		0.94 **	-0.30 **		0.37 **	0.45 **	0.46 **	0.42 **	0.51 **	0.19 *	0.34 **		0.57 ± 0.03	0.61 ± 0.02
**LWI**			0.30 **	0.27 **		0.50 **	0.20 *	0.19 *	0.86 **		-0.36 **		0.41 **	0.50 **	0.50 **	0.47 **	0.58 **		0.31 **		0.59 ± 0.08	0.63 ± 0.07
**PL**			0.18 **	0.33 **	-0.15 *	0.32 **	0.17 **	0.17 **	0.29 **	0.25 **		0.20 *	-0.16 *	-0.26 **		-0.26 **	-0.21 *	0.39 **		0.27 **	0.76 ± 0.03	0.70 ± 0.04
**PWI**		-0.32 **	0.37 **	0.18 *	-0.15 *	0.45 **	0.25 **	0.24 **	0.26 **	0.20 **	0.40 **		0.27 **	0.34 **	0.58 **	0.20 *	0.42 **				0.69 ± 0.04	0.75 ± 0.04
**PT**		-0.27 **	0.28 **		-0.21 **	0.37 **	0.21 **	0.27 **	0.20 **	0.18 **	0.14 *	0.52 **		0.42 **	0.34 **	0.44 **	0.46 **		0.21 *		0.46 ± 0.09	0.65 ± 0.03
**SL**		-0.20 **	0.32 **	0.24 **	-0.28 **	0.47 **		0.12 *	0.36 **	0.27 **	0.43 **	0.47 **	0.26 **		0.68 **	0.55 **	0.75 **	-0.27 **			0.89 ± 0.01	0.72 ± 0.03
**SWI**		-0.25 **	0.41 **	0.24 **	-0.22 **	0.54 **	0.22 **	0.23 **	0.40 **	0.30 **	0.41 **	0.64 **	0.31 **	0.78 **		0.49 **	0.72 **		0.17 *		0.86 ± 0.01	0.77 ± 0.04
**ST**		-0.25 **	0.22 **			0.33 **			0.21 **	0.12 *		0.40 **	0.30 **	0.45 **	0.46 **		0.75 **				0.83 ± 0.02	0.81 ± 0.02
**SW**		-0.24 **	0.43 **	0.22 **	-0.25 **	0.56 **	0.14 *	0.20 **	0.43 **	0.30 **	0.41 **	0.55 **	0.33 **	0.80 **	0.80 **	0.61 **		0.22 **	0.36 **		0.84 ± 0.02	0.77 ± 0.09
**NSP**			0.26 **	0.29 **		0.44 **	0.26 **	0.32 **	0.34 **	0.32 **	0.42 **	0.30 **	0.34 **		0.27 **				0.46 **	0.39 **	0.67 ± 0.04	0.55 ± 0.04
**NPP**			0.30 **	0.50 **		0.49 **	0.29 **	0.30 **	0.40 **	0.36 **	0.27 **	0.35 **	0.29 **	0.27 **	0.34 **	0.17 **		0.50 **		0.63 **	0.64 ± 0.05	0.57 ± 0.03
**SY**	0.15 *		0.37 **	0.57 **		0.51 **	0.34 **	0.35 **	0.44 **	0.40 **	0.36 **	0.32 **	0.23 **	0.35 **	0.41 **	0.16 *	0.44 **	0.44 **	0.87 **		0.55 ± 0.06	0.57 ± 0.09

*, ** significant at the 0.01 and 0.001 probability levels, respectively.

FT: flowering time; PGT: pod green time; PST: physiological maturity time; LMS: length of main stem; NPB: the number of primary stem branches; LI: internode length; BL: bracteole length; BWI: bracteole width; LL: leaflet length; LWI: leaflet width; PL: pod length; PWI: pod width; PT: pod thickness; SL: seed length; SWI: seed width; ST: seed thickness; SW: 100 seed weight; NSP: number of seeds per pod; NPP: number of pods per plant; SY: seed yield.

In order to maintain a good overview, only significant correlations are shown.
