# Supplementary material for: Major Contribution of Flowering Time and Vegetative Growth to Plant Production in Common Bean As Deduced from a Comparative Genetic Mapping
Source: Front Plant Sci. 2016 Dec 26;7:1940. doi: 10.3389/fpls.2016.01940 (PMC5183638; doi:10.3389/fpls.2016.01940)
Supplement: Supplementary file 5 [file Table5.PDF]

Supplementary Table 5. Linkage map constructed from the MA RIL population.

Linkage groups	Map length (cM)	No. of markers	Marker density (cM/marker)	Marker types			
				SCAR	SSR	SNP	*FIN*
1	94.4	15	6.3	-	14	-	1
2	127.7	24	5.3	-	23	1	-
3	109.3	20	5.5	-	20	-	-
4	116.4	10	11.6	-	10	-	-
5	112	7	16	-	7	-	-
6	104.3	15	6.9	-	15	-	-
7	134.1	14	9.6	-	14	-	-
8	96.8	16	6	1	15	-	-
9	118.2	16	7.4	-	16	-	-
10	70.6	13	5.4	-	12	1	-
11	105.1	16	6.6	-	12	4	-
**Total**	**1188.9**	**166**	**7.2**	**1**	**158**	**6**	**1**

.
