# Supplementary material for: Major Contribution of Flowering Time and Vegetative Growth to Plant Production in Common Bean As Deduced from a Comparative Genetic Mapping
Source: Front Plant Sci. 2016 Dec 26;7:1940. doi: 10.3389/fpls.2016.01940 (PMC5183638; doi:10.3389/fpls.2016.01940)
Supplement: Supplementary file 6 [file Table6.PDF]

Supplementary Table 6. Linkage map constructed from the AM RIL population.

Linkage groups	Map length (cM)	No. of markers	Marker density (cM/marker)	Marker types	
				SSR	*FIN*
1	61.1	18	3.4	17	1
2	140.9	26	5.4	26	-
3	92	21	4.4	21	-
4	138.6	11	12.6	11	-
5	79.8	10	8	10	-
6	90.4	15	6	15	-
7	109.2	15	7.3	15	-
8	144.4	16	9	16	-
9	131.6	22	6	22	-
10	139.3	15	9.3	15	-
11	48.2	11	4.4	11	-
**Total**	**1175.5**	**180**	**6.9**	**179**	**1**

.
