# Supplementary material for: Major Contribution of Flowering Time and Vegetative Growth to Plant Production in Common Bean As Deduced from a Comparative Genetic Mapping
Source: Front Plant Sci. 2016 Dec 26;7:1940. doi: 10.3389/fpls.2016.01940 (PMC5183638; doi:10.3389/fpls.2016.01940)
Supplement: Supplementary file 7 [file Table7.PDF]

Supplementary Table 7. Marker position on the genetic map and physical map on the reference genome.

Marker	Pv	CONSENSUS MAP	MA MAP	AM MAP	Chr	Physical position (Mb)
		Genetic position (cM)	Genetic position (cM)	Genetic position (cM)		
PVESTBR075	1	0		0	1	0.42
IAC049	1			6.94	1	1.74
PVESTBR271	1			14.94	1	1.83
BMc313	1			20.18	1	2.56
PVESTBR233	1			29	1	3.42
PVESTBR068	1	8.1	49.65		1	6.89
PVESTBR081	1	10.18	3.97		1	16.67
PVESTBR053	1	13.77	0	46.94	1	23.28
IAC076	1	30.81	21.86	37.37	1	26.45
IAC089	1	40.01	32.89		1	33.43
PvM120	1	45.67	40.34	25.36	1	3.42
BMB513	1	51.35	53.78		1	34.97
BMB290	1	59.96	47.59	36.15	1	36.04
PvM123	1		49.76		1	38.93
PVESTBR044	1	63.32		40.53	1	40.23
BMB502	1	67.44	57.02		1	40.23
BMB083	1	69.74		51.86	1	41.48
BMd045	1	73.17	62.74	56.01	1	44.97
FIN	1	76.93	66.92	57.88	1	45.56
PVESTBR268	1	78.65		61.15	1	47.68
BMc224	1	84.9	75.08	37.69	1	48.11
IAC023	1	93.91	83.05	43.25	1	50.72
BMB356	1	98.55	86.69	48.47	1	51.41

BMc324	1	105.13	94.38		1	49.04
IAC006	2	0	0	0	2	1.3
BMc210	2	2.84	3.65	1.44	2	1.78
BMd002	2	9.12	7.72	7.12	2	27.6
BM156	2	10.2		8.73	2	2.8
PVESTBR006	2	15.7	12.03	15.97	2	2.8
BM152	2		16.45		2	2.86
GATS91	2	18.97	20.56	16.08	2	3.89
IAC051	2	20.49		24.02	2	4.1
BMB527	2	22.79		27.67	2	10.38
BMB497	2	24.3	24.11		2	12.15
PVESTBR030	2	26.97	28.22		2	13.12
BMB097	2	28.36		31.75	2	13.65
BMB080	2	32		36.62	2	18.55
PVESTBR150	2	34.26	32.29	40.27	2	19.16
BMd017	2	39.52	36.19		2	21.97
BMc123	2	46.17	44.8	42.11	2	22.12
PVESTBR001	2	47.07		47.84	2	22.12
BMc367	2	52.51	49.37		2	22.12
PVESTBR164	2	58.74		67.69	2	27.03
PvM115	2	59.46	55.26	69.78	2	27.03
IAC013	2	64.21	67.16	76.7	2	32.03
BM221	2	72.51	73.94	78.63	2	34.21
PVESTBR083	2	76.09	61.88		2	37.47
BMd007	2		79.52		2	27.57
BMB469	2	84.8	87.83	90.34	2	40
BMc190	2	90.82		95.02	2	41.35
LEG-735	2	101.33	98.96		2	41.54

BMc280	2	106.75	106.21	103.22	2	41.88
PVESTBR008	2	112.7	110.39	109.53	2	41.88
PVESTBR046	2		110.38		2	45.07
BM172	2	122.76	117.23	135.62	2	45.31
BM237	2	125	119.58	135.72	2	45.31
PVESTBR218	2	128.32		140.92	2	47.3
BM164	2	130.96	127.71	136.81	2	45.48
IAC083	3			0	3	0.06
BMc122	3	0	0		3	1.63
PvM82	3	6.79	6.78		3	1.7
PVESTBR246	3			7.35	3	5.15
PVESTBR022	3	12.53	12.54		3	2.37
BMB506	3			14.3	3	11.55
BMc223	3	17.38	17.41		3	2.68
BMc259	3			18.6	3	12.29
BMc201	3			24.17	3	13.1
PvM148	3	28.34	28.42		3	5.15
BMB506	3	36.64	37.04		3	11.55
BMc259	3	41.79	41.95		3	12.29
BMc201	3	50.24	45.35		3	13.1
IAC024	3	61.33	51.2	29.32	3	19.09
BM187	3			31.91	6	20.25
PVESTBR230	3	62.55		36.8	3	24.83
BMB339	3			40.05	3	27.65
IAC056	3		52.54	40.05	3	28.53
BMB247	3	58.31		43.07	3	30.88
PvM126	3	64.94		45.33	3	32.93
IAC032	3	67.11	66.13	47.91	3	35.67

PvM81	3	78.05	67.19	53.96	3	38.75
BM098	3	87.75	74.58	55.25	3	39.44
BMc354	3			56.87	3	39.83
BMc180	3		79.77	66.99	3	44.43
BM282	3	88.15	83.04		3	45.78
BMd036	3	99.41	90.26	68.96	3	46.37
BM189	3	106.56	95.82	76.79	3	47.39
IAC081	3	112.35	103.06		3	48.33
BMB590	3	118.75	109.36	82.87	3	50.9
BMc298	3	123.4		89.41	3	51.99
PVESTBR131	3	125.85		91.97	3	52.02
PVESTBR193	4			0	4	0.4
BMc255	4	0	0	2.74	4	1.43
PVESTBR033	4	2.95	2.26		4	2.95
X04001	4	14.29	16.68	14.12	4	4.29
PVESTBR029	4	29.11		35.57	4	9.11
BMB066	4	36.07		50.9	4	26.07
BMB133	4		31.08		4	28.42
BMd055	4	58.98	52.03	63.67	4	28.98
BMB571	4	77.13	80.85	83.99	4	37.13
BM149	4		91.11		2	32.88
BMd026	4	91.49	102.72	89.93	4	41.49
BMd016	4	94.03			4	44.03
BMd009	4		111.46	110.25	4	44.02
BMc168	4	104.4		119.33	4	44.4
IAC052	4	115.45	116.45	138.57	4	45.45
IAC044	5	0	0	0	5	1.6
IAC010	5	3.69	5.54	2.69	5	2.69

IAC096	5	6.21		5.31	5	3.21
PVESTBR078	5	29.64		19.85	5	29.63
PVESTBR241	5	33.52		22.87	5	30.52
BM142	5	42.66	56.41	32.66	5	32.66
BM175	5	55.76	61.25	42.8	5	35.76
BMd020	5	68.2	85.8	55.47	5	38.2
PvM62	5	78.46	96.89	65.58	5	38.46
BMd028	5	89.92	111.96	79.8	5	39.92
BMc230	6	0	0	0	6	0.23
BMc284	6	1.01			6	6.19
BMc238	6	3.78		5.19	6	6.38
IAC047	6	9.58	0.93	12.64	6	8.14
BMB182	6	15.77	12.06	12.64	6	10.22
BMB341	6		21.96	16.8	6	13.48
BMc166	6		89.97	90.43	6	27.71
IAC001	6	19.51	36.02	17.21	6	20.08
BMB419	6	20.37		19.51	6	20.69
BMd011	6	27.52	43.16	26.35	6	21.12
BM284	6	32.93	47.83		6	21.49
PVESTBR072	6	38.88	51.48	33	6	24.81
BM170	6	54.53	66.56	57.68	6	25.09
PvBR14	6	65.13	80.16	63.16	6	27.66
PvBR20	6	66.99	76.94	84.87	6	27.66
BM275	6	68.73	79.15	64.4	6	27.66
BMc335	6	74.38	94.58		6	30.57
BMc161	6	84.71	104.29	72.3	6	31.83
BMB489	7	0	0	0	7	0.49
BM291	7	1.45	1.11		7	2.07

PVESTBR043	7	5.13	4.9		7	3.35
PVESTBR158	7	16.46	13.39	12.12	7	9.32
BMB202	7	18.66	16.42	15.35	7	13.82
PVESTBR025	7	23.22	22.59	16.46	7	14.18
IAC016	7	27.78	24.52	20.19	7	17.67
BMB621	7	31.86	29.29	23.21	7	18.29
BM210	7	38.02		29.83	7	31.12
PVESTBR119	7	45.5	51.47	39.77	7	31.85
PVESTBR034	7	55.44	61.27		7	43.3
BMc294	7	62.98	69.65	41.6	7	43.75
BMc248	7	64.53		44.87	7	43.91
PVESTBR279	7	67.15		47.65	7	45.55
IAC005	7	70.42		50.86	7	46.9
PVESTBR055	7		86.84		7	48
BMd040	7	76		56.71	7	49.36
BMc256	7	83.61	102.23	106.09	7	50.54
PVESTBR151	7	106.4	134.09	109.18	7	51.7
BMd025	8	0	0	0	8	0.93
PVESTBR107	8			11.98	8	1.54
IAC092	8		1.67		8	5.27
SC08	8		20.55		8	7.41
BM165	8	17.85	30.69	24.75	8	7.85
PVESTBR010	8	18.85		31.89	8	8.85
BMB445	8	30.2	31.29	41.66	8	10.2
BM151	8			57.91	8	10.39
BM211	8	40.71	43.77	65.05	8	10.71
BM153	8	53.32	45.73	72.19	8	13.32
BMB267	8	55.87			8	15.87

BMB174	8	62.97	49.96		8	22.97
BM238	8	68.49	52.35	78.13	8	23.49
IAC074	8	73.6	57.53	85.27	8	43.6
BMc353	8	75.12	60.23	92.41	8	45.12
PVESTBR106	8			105.18	8	45.12
BM167	8			108.42	8	47.87
BMc316	8	88.34	62.63	119.64	8	48.34
IAC022	8			132.41	8	52.79
BMc182	8		144.39		8	54.95
BMB559	8	92.58			8	52.58
BMB474	8		69.36		8	54.72
PVESTBR204	8	105.75			8	55.74
BM224	8	107.65	73.33		8	57.65
PVESTBR098	8	108.98	86.82		8	57.98
IAC102	8	109.08	96.78		8	59.08
PvM73	9	0	0	0	9	0.85
PVESTBR070	9	5.92		11.19	9	2.14
BMB461	9	7.65	4.92	17.03	9	7.48
PvM128	9	14.52	13.9	23.25	9	8.09
BM272	9	24.01	22.43	26.6	9	9.33
IAC062	9	29.41	28.32	29.84	9	10.2
BMc184	9	33.65	32.21	35.52	9	10.56
BM141	9	44.58	40.83	47.17	9	14.25
BMd054	9		52.45		9	17.48
Pvtttc001	9		60.49	53.32	9	17.97
PVESTBR082	9	45.29		58.77	9	18.26
BM154	9	50.16		63.84	9	1.85
IAC068	9	58.62			9	20.56

BMc327	9	58.69		70.3	9	22.9
BMB264	9	61.76		78.98	9	25.54
PVESTBR073	9	73.96	62.47	83.68	9	26.61
BM202	9	76.26	64.51	85.8	9	26.63
IAC033	9	80.22	69.23		9	28.89
IAC058	9	85.98	75.44	92.69	9	28.89
BMc221	9	92.92	83.11	94.47	9	31.42
BMB493	9	108.33	100.64	105.76	9	33.88
BMB563	9	122.67		120.08	9	35.12
PVESTBR166	9	125.1		122.7	9	36.15
BMc318	9	129.87	118.25	127.1	9	36.56
IAC019	9	134.3		131.65	9	37.36
SNP-5459	10	0	9.29		10	5.7
BMB96	10	3.39	12.87	5.37	10	6.64
BMc234	10	5.9	15		10	6.87
BM157	10	8.9	18.33	6.24	10	8.99
BMc159	10	10.67	22.7		10	35.16
GATS11B	10	11.73	21.4	87.99	10	32.69
BMB096	10	12.27			10	29.83
BMB106	10	13.86	20.29	82.05	10	29.83
BMB302	10	17.34	0	0	10	2.08
PVESTBR017	10	19.95	29.74		10	39.06
PVESTBR049	10	25.17		99.97	10	38.41
BM229	10	25.37		11.61	10	10.14
PVESTBR016	10	33.81	39.94	108.39	10	39.1
BMB447	10	36.09		54.01	10	18.15
BM277	10	40.06		68.46	10	24.89
BMB221	10	43.53		39.57	10	18.04

BMc150	10	47.41	70.63	128.78	10	42.29
BMB152	10	51.98		29.8	10	16.15
X63525	10	53.83	55.38	116.8	10	40.68
BM212	10	56.54		139.26	10	43.2
PVESTBR003	10	68.18	67.85		10	41.8
PvM152	11	0	0		11	1.6
BM240	11	10.17	10.1	0	11	1.61
PVESTBR057	11	18.36	22.39	1.74	11	2.85
IAC075	11		25.22		11	39.65
BMd033	11	28.21	33.86	6.48	11	3.47
BMc307	11	37.19	46.99	7.97	11	4.41
PVESTBR087	11	44.46	48.24		11	6.44
IAC004	11		69.5	9.24	3	25.3
BMc322	11	51.86	74.38	18.5	11	19.86
PVESTBR013	11	52.21	75.05		11	19.86
SNP-5101	11		79.67		11	25.89
SNP-3839	11	57.9	82.37		11	26.57
SNP-3273	11	61.96	86.18		11	29.71
PVESTBR152	11		90.24		9	17.49
SNP-3616	11	66.03	92.46		11	37.24
PvM98	11	68.94	96.37	26.76	11	38.01
BMB428	11	78.46		32.04	11	43.06
BMB619	11	83.71		44.66	11	46.86
PVESTBR071	11	86.29	105.07	48.22	11	49.71

.
