# Supplementary material for: Major Contribution of Flowering Time and Vegetative Growth to Plant Production in Common Bean As Deduced from a Comparative Genetic Mapping
Source: Front Plant Sci. 2016 Dec 26;7:1940. doi: 10.3389/fpls.2016.01940 (PMC5183638; doi:10.3389/fpls.2016.01940)
Supplement: Supplementary file 8 [file Image1.PDF]

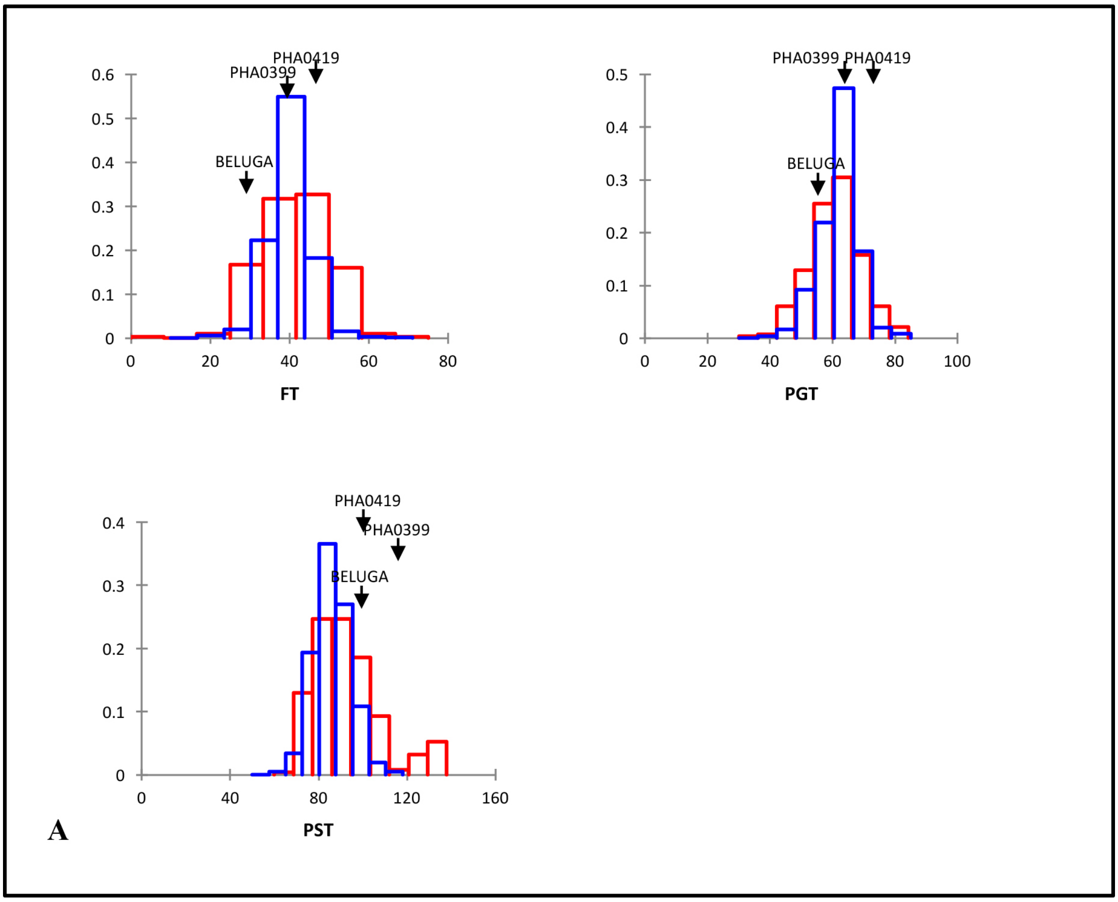

PHA0419
PHA0399
BELUGA
FT
PHA0399 PHA0419
BELUGA
PGT
PHA0419
PHA0399
BELUGA
PST
A

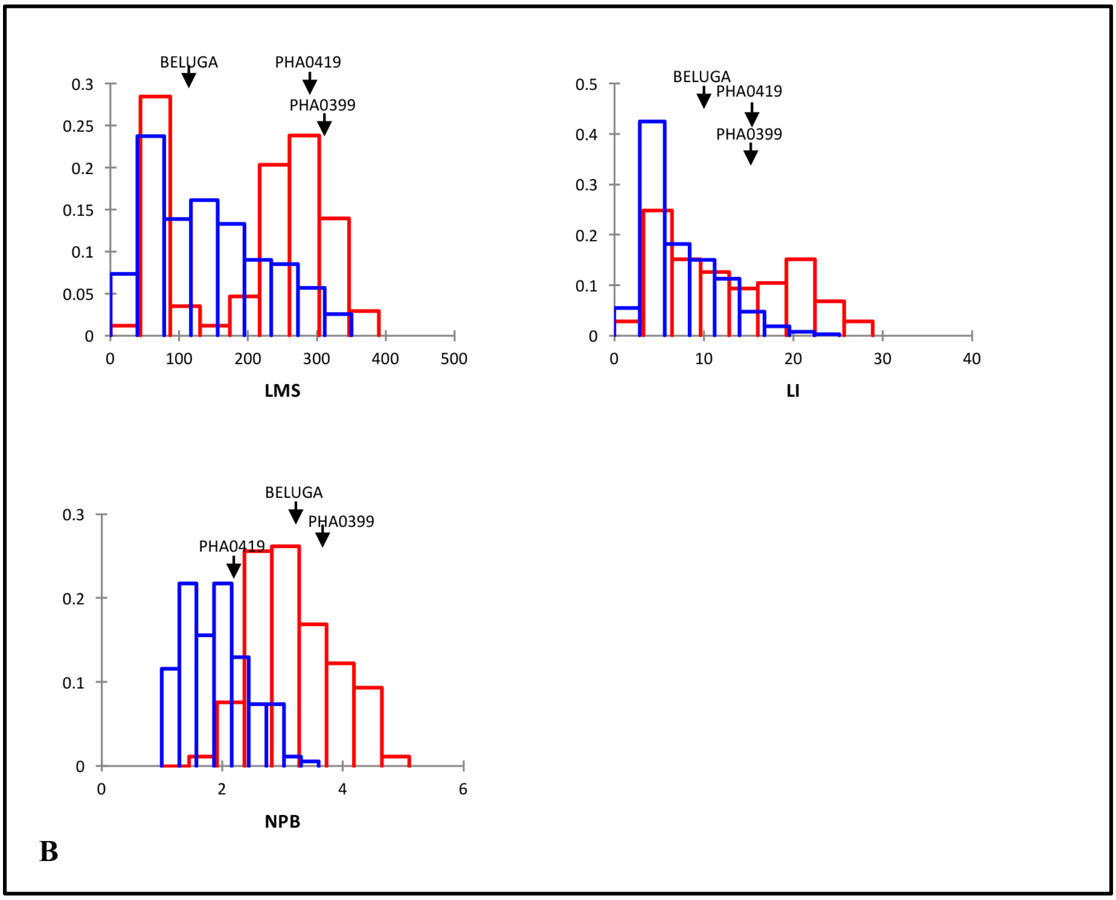

BELUGA
PHA0419
PHA0399
LMS
BELUGA
PHA0419
PHA0399
LI
BELUGA
PHA0399
PHA0419
NPB
B

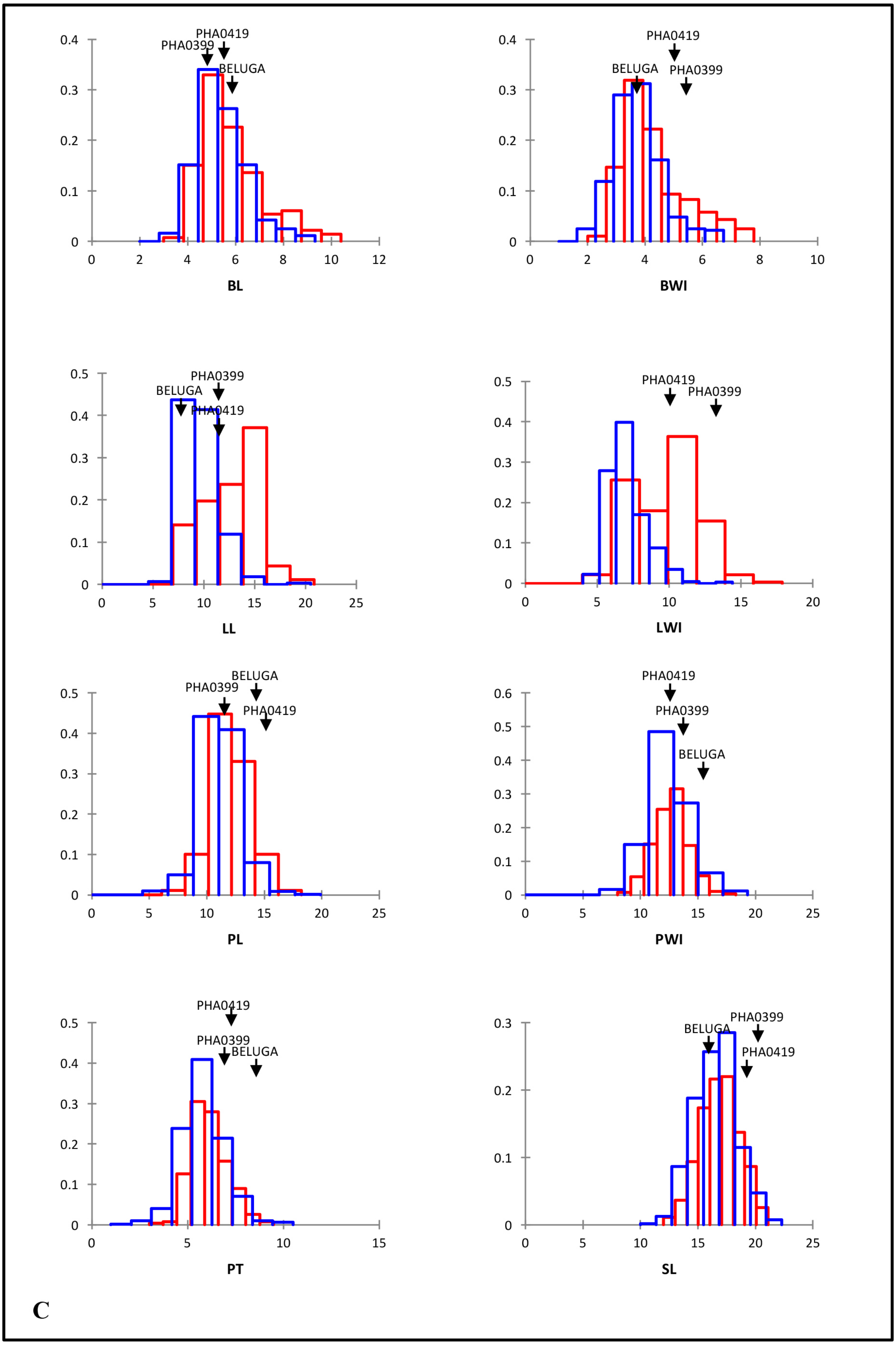
PHA0419
PHA0399
BELUGA
BL
BWI
LL
LWI
PL
PWI
PT
SL
C

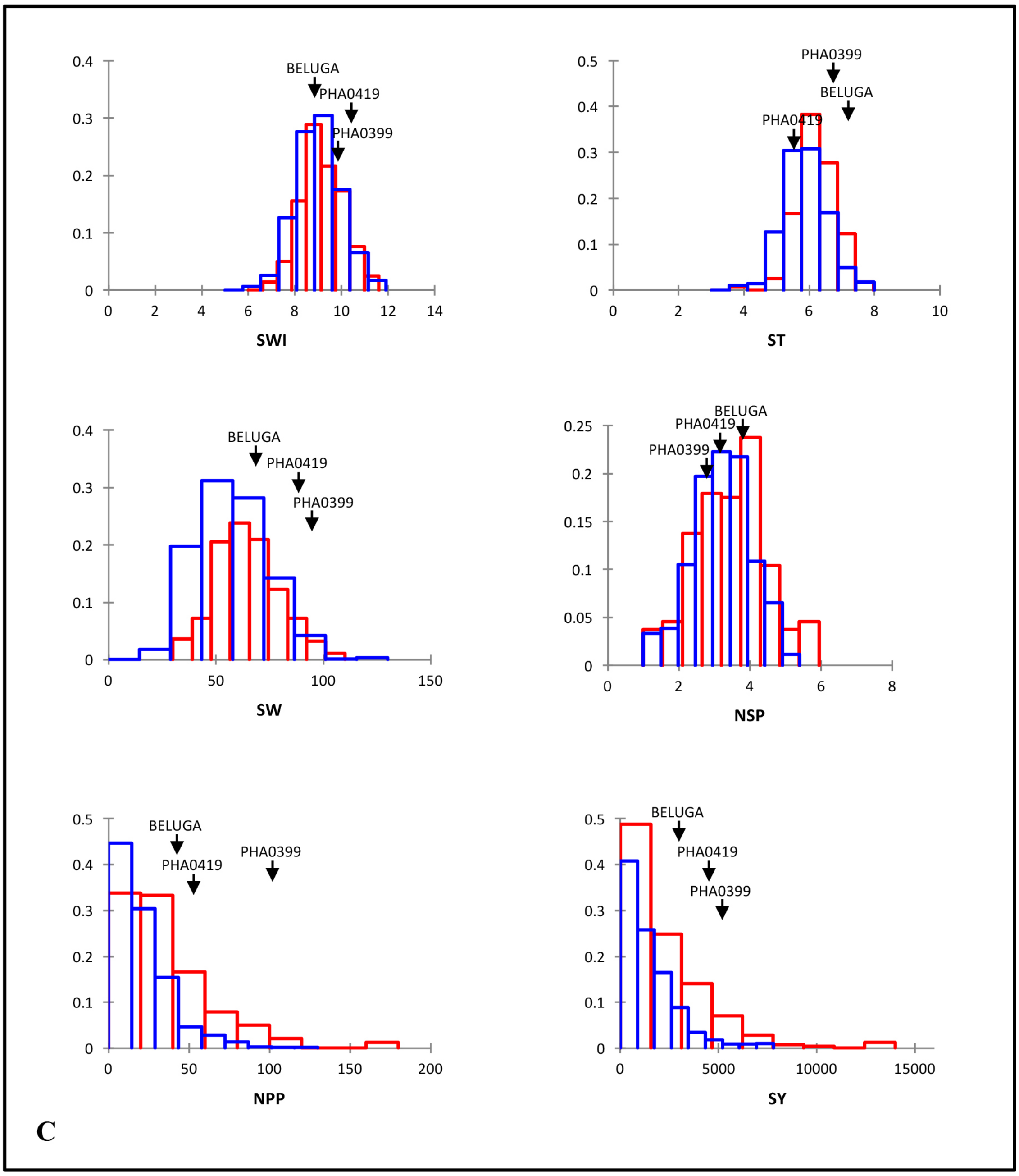

Figure S1. Phenotypic distribution of the RIL population based on line means for flowering and maturity traits (A): days to flowering (FT), days to immature pod harvest (PGT) and days to physiological pod maturity (PST); vegetative growth traits (B): length of main stem plant height (LMS), the number of primary stem branches (NPB) and internode length (LI); and plant production traits (C): bracteole length (BL), bracteole width (WI), leaflet length (LL), leaflet width (LWI), pod length (PL), pod width (PWI), pod thickness (PT), seed length (SL), seed width (SWI), seed thickness (ST), 100 seed weight (SW), number of seeds per pod (NSP), number of pods per plant (NPP), and seed yield (SY). Horizontal axis is means of MA and AM RIL populations (blue and red lines, respectively) grown in four environments. Vertical axis is relative frequency. Arrows indicate the mean values of the parental lines PHA0419, PHA0399 and Beluga.
